# Supplementary material for: Inflammatory Signaling by NOD-RIPK2 Is Inhibited by Clinically Relevant Type II Kinase Inhibitors
Source: Chem Biol. 2015 Sep 17;22(9):1174–84. doi: 10.1016/j.chembiol.2015.07.017 (PMC4579271; doi:10.1016/j.chembiol.2015.07.017)
Supplement: Document S1. Figures S1–S7, Tables S1 and S2, and Supplemental Experimental Procedures [file mmc1.pdf]

Chemistry & Biology, Volume *22*

Supplemental Information

Inflammatory Signaling by NOD-RIPK2 Is Inhibited by Clinically Relevant Type II Kinase Inhibitors

Peter Canning, Qui Ruan, Tobias Schwerd, Matous Hrdinka, Jenny L. Maki, Danish Saleh, Chalada Suebsuwong, Soumya Ray, Paul E. Brennan, Gregory D. Cuny, Holm H. Uhlig, Mads Gyrd-Hansen, Alexei Degterev, and Alex N. Bullock

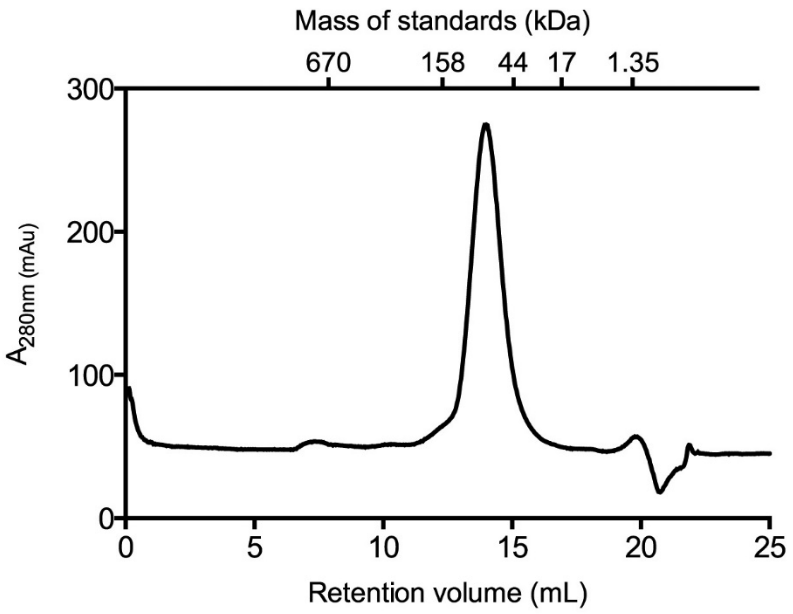

Figure S1, Related to Figure 2. Dimerization of RIPK2 in solution. Analytical gel filtration trace of RIPK2. Some 500 µL of purified RIPK2 at 50 µM concentration was injected onto a superdex 200 10/300 GL column and monitored by absorbance at 280 nm (A280). The retention volume of RIPK2 was compared with the retention volumes of gel filtration MW standards and indicated that the protein is a dimer in solution.

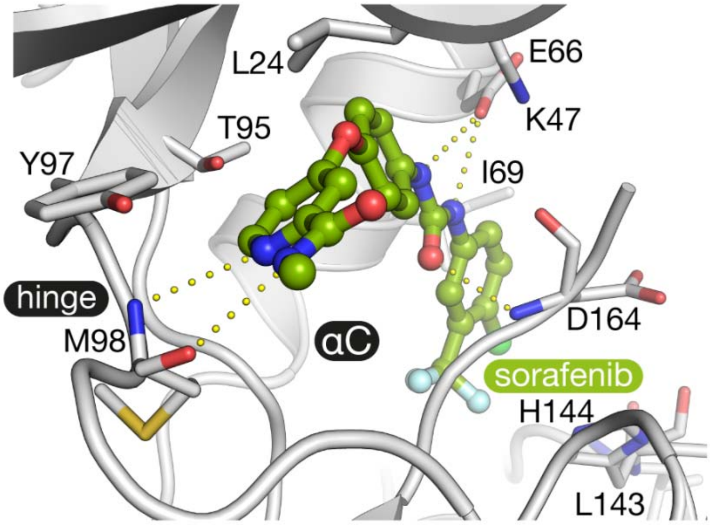

Figure S2, Related to Figure 3. Predicted binding mode of sorafenib. The main chain is shown as a cartoon representation and the side-chains of selected residues shown as sticks with residue numbers. Hydrogen bonds formed between RIPK2 and bound inhibitor are shown as dashed lines. Docking was performed with ICM-Pro (Molsoft).

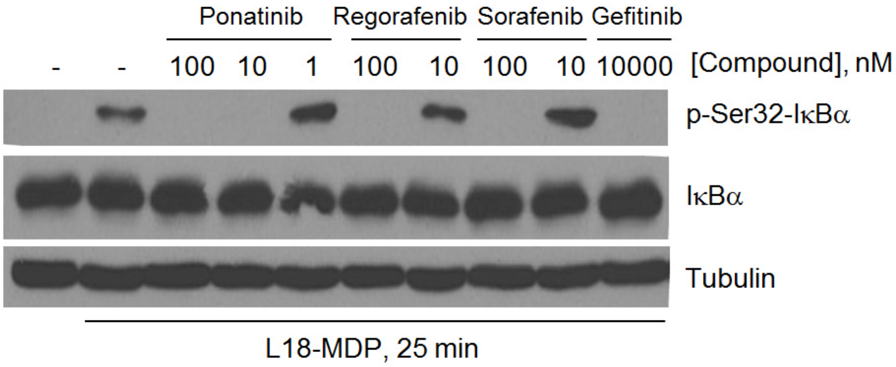

Figure S3, Related to Figure 4. Inhibitors of RIPK2 block IκBα phosphorylation in a dose-dependent manner. Phosphorylation of IκBα in HEKBlue cells. Cells were treated with indicated concentrations of inhibitors, followed 30 minutes later by stimulation with 1 μg/mL L18-MDP. Cells were harvested after 25 minutes and changes in IκBα protein phosphorylation were analyzed by Western blotting. Levels of tubulin were used as a loading control.

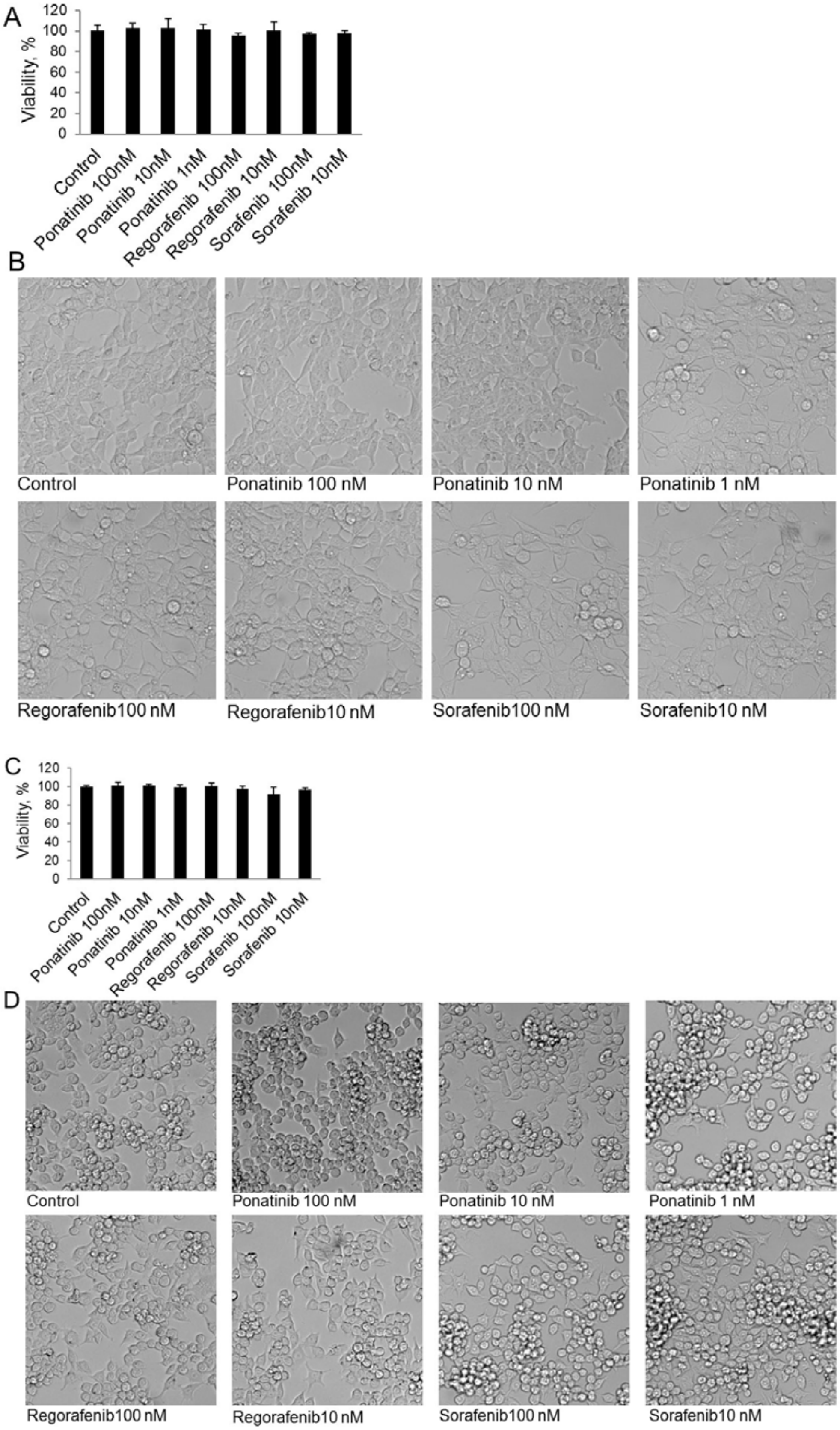
A
Viability, %
120
100
80
60
40
20
0
Control
Ponatinib 100nM
Ponatinib 10nM
Ponatinib 1nM
Regorafenib 100nM
Regorafenib 10nM
Sorafenib 100nM
Sorafenib 10nM
B
Control
Ponatinib 100 nM
Ponatinib 10 nM
Ponatinib 1 nM
Regorafenib100 nM
Regorafenib10 nM
Sorafenib100 nM
Sorafenib10 nM
C
Viability, %
120
100
80
60
40
20
0
Control
Ponatinib 100nM
Ponatinib 10nM
Ponatinib 1nM
Regorafenib 100nM
Regorafenib 10nM
Sorafenib 100nM
Sorafenib 10nM
D
Control
Ponatinib 100 nM
Ponatinib 10 nM
Ponatinib 1 nM
Regorafenib100 nM
Regorafenib10 nM
Sorafenib100 nM
Sorafenib10 nM

Figure S4, Related to Figure 4 and Figure 6. Inhibitors of RIPK2 do not impact cell viability or morphology. Cells were treated with the indicated concentrations of the inhibitors for 24 hours. (B) Cell viability in HEKBlue cells. (C) Morphology of HEKBlue cells. (D) Cell viability of RAW264.7cells. (E) Morphology of RAW264.7 cells. None of the inhibitors affected the viability or morphological appearance of RAW264.7 cells, except for 100 nM ponatinib, which caused rounding of RAW cells and likely reflects off target activity at the highest concentration. Experiments were performed in triplicate, error bars indicate SD values.

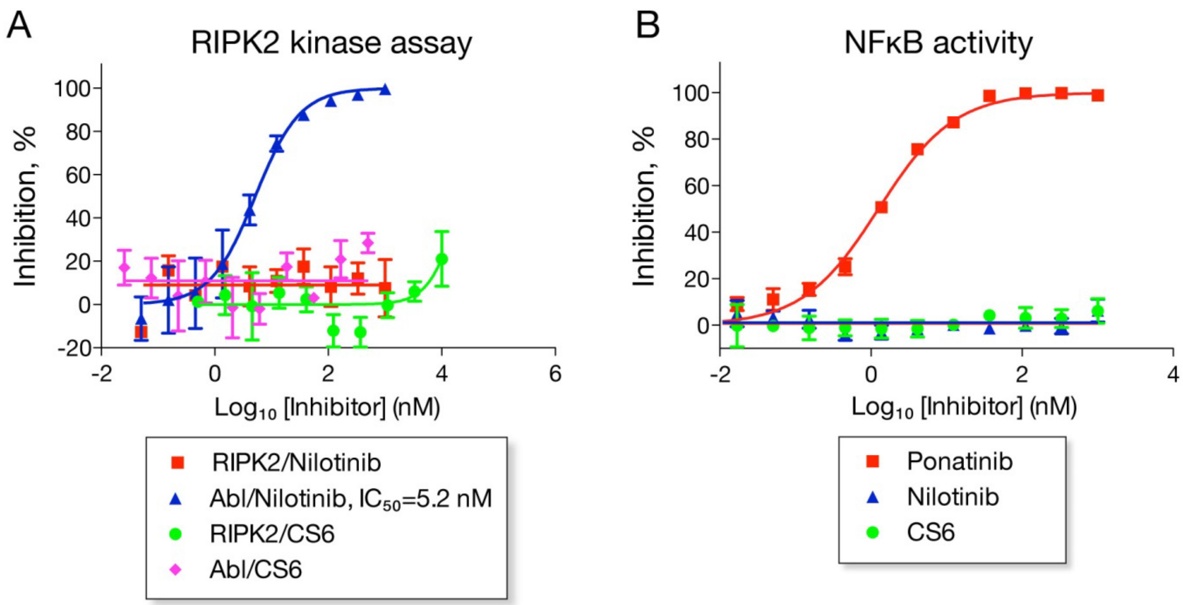

Figure S5, Related to Figure 3 and Figure 4. Analysis of Abl and RIPK2 inhibition by nilotinib and CS6. (A) Dose-response curves for RIPK2 inhibition by nilotinib and CS6. Experiments were performed in duplicate. *In vitro* kinase activity was measured using the ADPGlo assay. Non-linear curve fitting to calculate IC50 values was performed using Prism software. Experiments were performed in duplicate, error bars indicate SD values. (B) Inhibition of NFκB activation in HEKBlue cells. HEKBlue reporter cells, expressing NOD2 and NFκB-SEAP reporter were treated with 6-8 concentrations of each inhibitor in triplicate followed by stimulation with 1 μg/mL L18-MDP for 8 hours. SEAP activity was detected using HEKBlue media with detection of absorbance at 620 nM in a Wallac3V plate reader. Non-linear curve fitting to calculate EC50 values was performed using Prism software. Experiments were performed in triplicate, error bars indicate SD values.

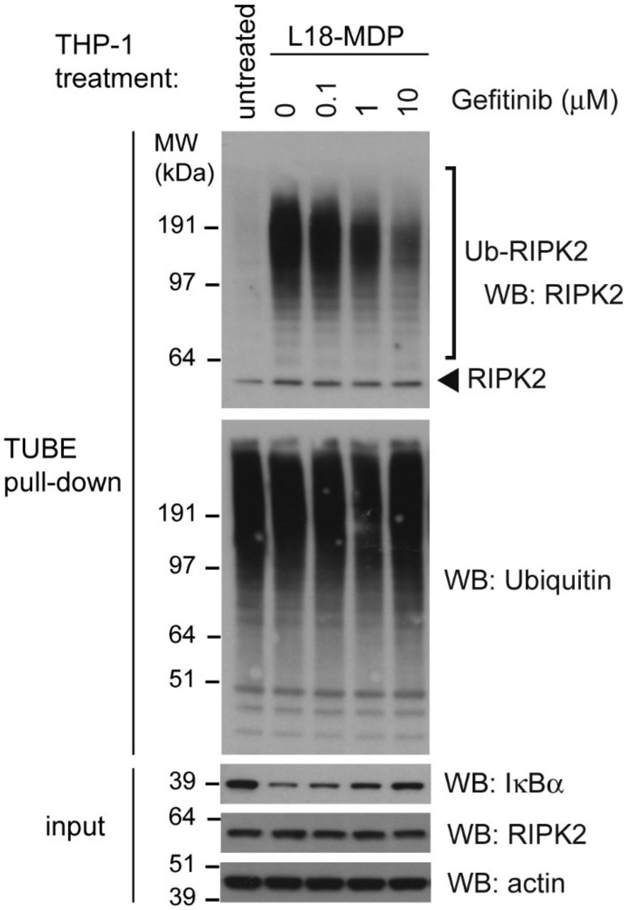

Figure S6, Related to Figure 5. Inhibition of NOD2-dependent ubiquitination. RIPK2 ubiquitination in THP-1 cells upon L18-MDP stimulation (1 hour) in the presence of increasing concentrations of gefitinib. Representative result of the experiment performed three times.

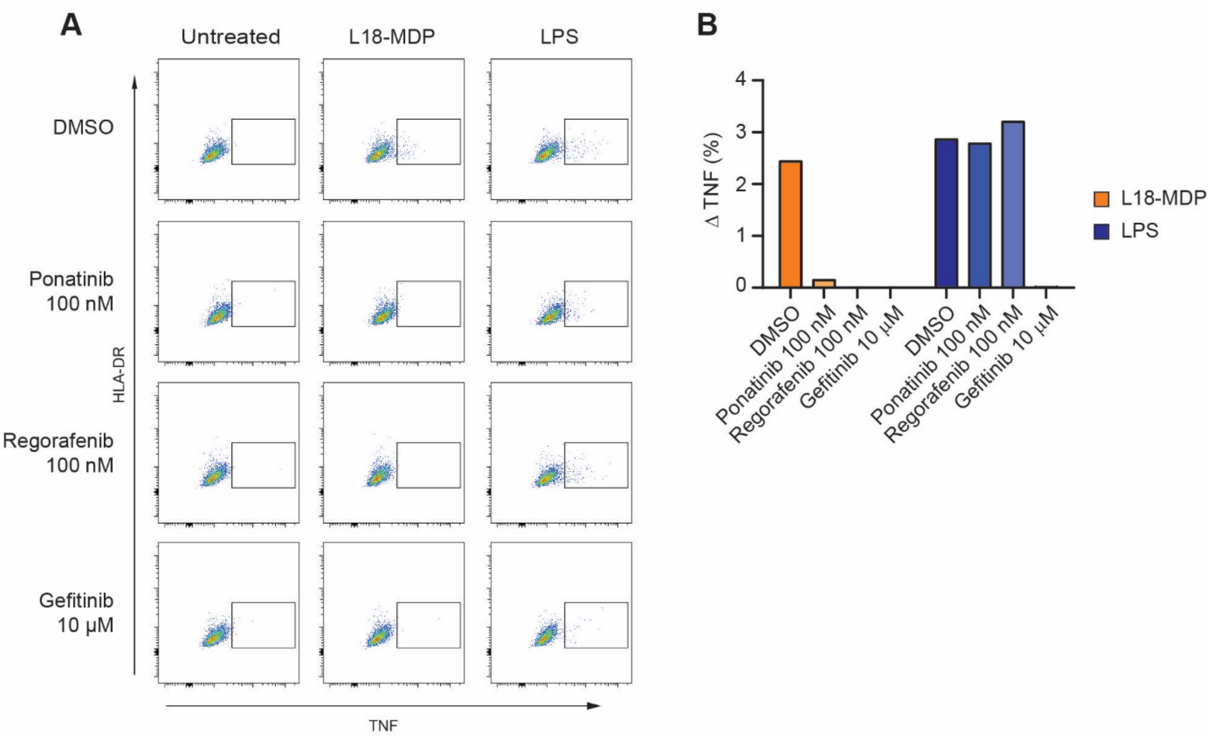

Figure S7, Related to Figure 7. Inhibition of NOD2 signaling in the human monocytic cell line THP-1. Representative FACS blots (A) and quantification of TNF production (B) in THP-1 cells stimulated with L18-MDP or LPS. In indicated conditions cells were preincubated with kinase inhibitors for 1 hour before activation of NOD2 and TLR4 receptor for 2.5 hours.

Supplemental Data

Table S1, Related to Figure 1 Thermal Shift Inhibitor Screening for RIPK2

Rank	Tm shift (°C)	Inhibitor	Supplier	Supplier I.D.
1	23.1	Ponatinib (AP24534)	Selleck Chemicals	S1490
2	12.1	LDN-193189	Paul Yu, Harvard	N/A
3	10.0	K252a	Calbiochem (EMD)	420298
4	9.6	Dorsomorphin	Sigma	P5499
5	9.5	Dasatinib	Sequoia	863127-77-9
6	9.5	Gefitinib	Biaffin GmbH	PKI-GFTB-010
7	9.1	Staurosporine	AXXORA	S-9300
8	8.9	Raf1 kinase inhibitor II	Calbiochem (EMD)	553011
9	8.7	BIBX1382	Calbiochem (EMD)	324832
10	8.4	TGFβ inhibitor IV (A-83-01)	Calbiochem (EMD)	616454

Table S2, Related to Figure 1 Diffraction data collection and refinement statistics

**Data collection**	
Wavelength (Å)	0.9795
Resolution range (Å)	58.74 - 2.75 (2.9 - 2.75)
Space group	$P2_12_12_1$
Cell dimensions	
a, b, c (Å)	58.7, 86.7, 137.3
α, β, γ (°)	90, 90, 90
Total reflections	136,896 (19978)
Unique reflections	18905 (2690)
Multiplicity	7.2 (7.4)
Completeness (%)	100 (100.00)
Mean $(I)/\sigma(I)$	12.1 (2)
Wilson *B*-factor ($Å^2$)	63.32
$R_{p.i.m}$	0.066 (0.557)
$CC_{1/2}$	0.998 (0.759)
**Refinement**	
R-work	0.2006 (0.297)
R-free	0.2436 (0.295)
Number of atoms	4445
macromolecules	4318
ligand	78
water	45
Protein residues	560
R.M.S. deviations	
Bond lengths (Å)	0.01
Bond angles (°)	1.295
Ramachandran favoured (%)	96
Ramachandran outliers (%)	0
MolProbity clashscore	4.58
Avg *B*-factors ($Å^2$)	63.7
macromolecules	64.2
solvent	48.6
ligand	46

Values in brackets show the statistics for the highest resolution shells.
R.M.S indicates root-mean-square.

Supplemental Experimental Procedures

Antibodies and Reagents

Commercially available antibodies for immunoblotting were obtained from the following sources: mouse anti-RIPK1 (clone 38/RIP) from BD BioSciences, mouse anti-RIPK2 (clone A-10) and rabbit RIPK2 (clone H-300) from Santa Cruz, mouse anti-Ubiquitin (clone Ubi-1, Imgenex), rabbit anti-phospho-Ser176-RIPK2 (Cell Signaling), mouse anti-α-tubulin (clone DM1A, Cell Signaling), rabbit anti-phospho-Thr202/Tyr204 Erk1/2 (clone D13.14.4E, Cell Signaling), mouse anti-phospho-Tyr (clone P-Tyr-100, Cell Signaling), mouse anti-IκBα (clone L35A5, Cell Signaling), and anti-actin (clone MAB1501, Millipore). The NOD1 ligand Tri-DAP, NOD2 ligands L18-MDP and MDP and TLR2 ligand Pam3CSK4 were purchased from Invivogen, LPS (*E.coli* 0111:B4) from Sigma and recombinant TNF from Enzo.

Cells

THP-1 cells were cultured in RPMI 1640 medium (Gibco) supplemented with 10% FBS and Penicillin / Streptomycin at density 0.6 −1.0 million cells per mL. RAW264.7 cells were maintained in DMEM medium (Fisher) supplemented with 10% FBS (Sigma) and 1% antibiotic-antimycotic mix (PSA) (Invitrogen). HEK-Blue hNOD2 cells (Invivogen) were maintained in DMEM/10% FBS/PSA supplemented with Normocin (100 µg/mL), Blasticidin (30 µg/mL) and Zeocin (100 µg/mL).

Western blot analysis

HEK-Blue cells were seeded into 10 cm^2 dishes to achieve 80-90% confluency after 48 hr. Cells were pre-treated with inhibitors for 30 min and stimulated with L18-MDP for 30 min. Cells were lysed in RIPA buffer supplemented with PMSF (Cell Signaling), briefly sonicated and spun in

4^0C centrifuge at 14,000 rpm for 15 min to collect lysates. Protein concentrations were measured using 660 nM protein assay reagent (Pierce). Equal amounts of proteins were separated using 8-10% SDS-PAGE, followed by overnight incubations with antibodies according to the manufacturer's recommendations.

ADPGlo *in vitro* kinase assays

For ADPGlo (Promega) assays, 1 ng Abl or 10 ng of RIPK2 was diluted in reaction buffer (40 mM Tris-HCl pH 7.5, 20 mM $MgCl_2$, 0.5 mM DTT, 0.01% BSA) supplemented with 50 μM ATP and 10-point dose range of inhibitors. Reactions were performed at room temperature for 1 hour. Reactions were performed in 5 μL total volume (5% final concentration of DMSO) and stopped by addition of 5 μL of ADPGlo reagent for 40 min at room temperature. Luminescent signal was generated by addition of 10 μL of kinase detection reagent for 30 minutes at room temperature and determined using Victor3V platereader (Perkin Elmer). Specific signal was calculated by subtracting values in the wells without protein and inhibitor from the values in the test wells. Inhibition, % = ((specific signal (DMSO control) - specific signal (inhibitor))/ (specific signal (DMSO control))) x 100%. Non-linear regression to determine IC_{50} values was performed using Prism software (GraphPad).

qPCR analysis in RAW264.7 cells

RAW cells were seeded at $2X10^5$ cells/well in 12 well plates. After 24 hours, cells were treated with inhibitors and stimulated with 10 μg/mL MDP for 24 hours. Total RNAs were isolated using ZR RNA miniprep kit. 1 μg of total RNA was used to synthesize cDNA using iScript cDNA synthesis kit (Bio-Rad). cDNAs were diluted 5-fold with water and 2 μL were used in 20 μL qPCR reactions including 10 μL 2X VeriQuest SYBR mix (Affymetrix) and 1 μL of 10 μM primers. Cycling parameters were: 50°C – 2 min, 95°C – 10 min, 45 cycles: 95°C – 15 sec,

60°C – 30 sec. Reactions were performed using a Roche480 machine. GAPDH was used as a house keeping control to normalize mRNA values. The following primers were used: *mGAPDH* forward 5'-TGTGTCCGTCGTGGATCTGA-3'; *mGAPDH* reverse 5'-GGTCCTCAGTGTAGCCCAAG-3'; *mCCL4* forward 5'-TTCCTGCTGTTTCTCTTACACCT-3'; *mCCL3* reverse 5'-CTGTCTGCCTCTTTTGGTCAG-3'; *mCXCL2* forward 5'-CCAACCACCAGGCTAGAGG-3'; *mCXCL2* reverse 5'-GCGTCACACTCAAGCTCTG-3'; *mRANTES* forward 5'-TTTGCCTACCTCTCCCTCG-3'; *mRANTES* reverse 5'-CGACTGCAAGATTGGAGCACT-3'.

Purification of RIPK2

DNA encoding the kinase domain of human *RIPK2* (Uniprot: O43353, residues 8-317) was cloned into the transfer vector pFB-LIC-Bse, which encodes for an N-terminal hexahistidine tag and a Tobacco Etch Virus Protease A (TEV) cleavage site. Bacmid DNA was prepared in *E. coli* strain DH10Bac and used to generate baculoviruses in Sf9 insect cells. For expression, viruses were used to Sf9 infect cells grown in Insect-Xpress media (Lonza) to a density of $2x10^6$ cells/mL. Cells were harvested after 48 hours by centrifugation and resuspended in 15 mL binding buffer (50 mM HEPES pH 7.4, 500 mM NaCl, 5% glycerol, 5 mM Imidazole) supplemented with protease inhibitor cocktail set V (Calbiochem) at 1:1000 dilution. Cells were lysed using an Emulsiflex C5 homogeniser, clarified by centrifugation, and the recombinant protein collected by nickel-affinity chromatography and eluted by imidazole. RIPK2 protein was treated with lambda phosphatase overnight at 4°C before further purification on a Superdex 75 26/60 gel filtration column pre-equilibrated in 10 mM HEPES pH 7.4, 500 mM NaCl, 5% glycerol, 1 mM tris(2-carboxyethyl)phosphine (TCEP). The final protein was supplemented with 5 mM L-arginine, 5 mM L-glutamate and 2 mM DTT. DNA sequencing and mass spectrometry identified an Arg171Cys mutation in the kinase activation loop.

Crystallization and structure determination

RIPK2 was concentrated to 3.7 mg/mL and ponatinib added to a slight molar excess. Crystals were grown using the vapour-diffusion technique in 150 nL sitting drops containing 50 nL protein and 100 nL of a reservoir solution containing 0.1 M ammonium citrate and 16% (w/v) PEG3350 at 20°C. Crystals were cryo-protected by addition of 25% ethylene glycol before being vitrified in liquid nitrogen. Diffraction data were collected at 100K on Diamond Light Source beamline I04. Data were indexed and integrated using XDS (Kabsch, 2010; Leslie and Powell, 2007) and scaled using AIMLESS (Evans and Murshudov, 2013) in the CCP4 suite of programs (Winn, et al., 2011). Phases were identified using molecular replacement in PHASER (McCoy, et al., 2007) and the PDB: 3PPZ as a search model. Structures were built using PHENIX.AUTOBUILD (Adams, et al., 2010) and then refined and modified using alternate rounds of REFMAC5 (Murshudov, et al., 2011) and COOT (Emsley, et al., 2010). TLS groups were determined using the TLSMD server (Painter and Merritt, 2006). The refined structure was validated with MolProbity (Chen, et al., 2010) and the atomic coordinate files deposited in the Protein Data Bank with Autodep (Yang, et al., 2004). Structure figures were prepared with PyMOL (Schrödinger LLC., Version 1.2r3pre), sequence alignments with ESPript (Robert and Gouet, 2014) and structure comparisons using PDBeFOLD (Krissinel and Henrick, 2004).

Supplemental References

Adams, P.D., Afonine, P.V., Bunkoczi, G., Chen, V.B., Davis, I.W., Echols, N., Headd, J.J., Hung, L.W., Kapral, G.J., Grosse-Kunstleve, R.W., et al. (2010). PHENIX: a comprehensive Python-based system for macromolecular structure solution. Acta Crystallogr D Biol Crystallogr 66, 213-221.

Chen, V.B., Arendall, W.B., 3rd, Headd, J.J., Keedy, D.A., Immormino, R.M., Kapral, G.J., Murray, L.W., Richardson, J.S., and Richardson, D.C. (2010). MolProbity: all-atom

structure validation for macromolecular crystallography. Acta Crystallogr D Biol Crystallogr 66, 12-21.

Emsley, P., Lohkamp, B., Scott, W.G., and Cowtan, K. (2010). Features and development of Coot. Acta Crystallogr D Biol Crystallogr 66, 486-501.

Evans, P.R., and Murshudov, G.N. (2013). How good are my data and what is the resolution? Acta Crystallogr D Biol Crystallogr 69, 1204-1214.

Kabsch, W. (2010). Xds. Acta Crystallogr D Biol Crystallogr 66, 125-132.

Krissinel, E., and Henrick, K. (2004). Secondary-structure matching (SSM), a new tool for fast protein structure alignment in three dimensions. Acta Crystallogr D Biol Crystallogr 60, 2256-2268.

Leslie, A.G.W., and Powell, H.R. (2007). Processing diffraction data with mosflm

Evolving Methods for Macromolecular Crystallography, R.J. Read, and J.L. Sussman, eds. (Springer Netherlands), pp. 41-51.

McCoy, A.J., Grosse-Kunstleve, R.W., Adams, P.D., Winn, M.D., Storoni, L.C., and Read, R.J. (2007). Phaser crystallographic software. J Appl Crystallogr 40, 658-674.

Murshudov, G.N., Skubak, P., Lebedev, A.A., Pannu, N.S., Steiner, R.A., Nicholls, R.A., Winn, M.D., Long, F., and Vagin, A.A. (2011). REFMAC5 for the refinement of macromolecular crystal structures. Acta Crystallogr D Biol Crystallogr 67, 355-367.

Painter, J., and Merritt, E.A. (2006). Optimal description of a protein structure in terms of multiple groups undergoing TLS motion. Acta Crystallogr D Biol Crystallogr 62, 439-450.

Robert, X., and Gouet, P. (2014). Deciphering key features in protein structures with the new ENDscript server. Nucleic Acids Res 42, W320-324.

Schrödinger LLC. (Version 1.2r3pre). The PyMOL Molecular Graphics System.

Winn, M.D., Ballard, C.C., Cowtan, K.D., Dodson, E.J., Emsley, P., Evans, P.R., Keegan, R.M., Krissinel, E.B., Leslie, A.G., McCoy, A., et al. (2011). Overview of the CCP4 suite and current developments. Acta Crystallogr D Biol Crystallogr 67, 235-242.

Yang, H., Guranovic, V., Dutta, S., Feng, Z., Berman, H.M., and Westbrook, J.D. (2004). Automated and accurate deposition of structures solved by X-ray diffraction to the Protein Data Bank. Acta Crystallogr D Biol Crystallogr 60, 1833-1839.
