# Supplementary material for: Inflammatory Signaling by NOD-RIPK2 Is Inhibited by Clinically Relevant Type II Kinase Inhibitors
Source: Chem Biol. 2015 Sep 17;22(9):1174–84. doi: 10.1016/j.chembiol.2015.07.017 (PMC4579271; doi:10.1016/j.chembiol.2015.07.017)
Supplement: Document S2. Article plus Supplemental Information [file mmc2.pdf]

Article

Chemistry & Biology

Inflammatory Signaling by NOD-RIPK2 Is Inhibited by Clinically Relevant Type II Kinase Inhibitors

Graphical Abstract

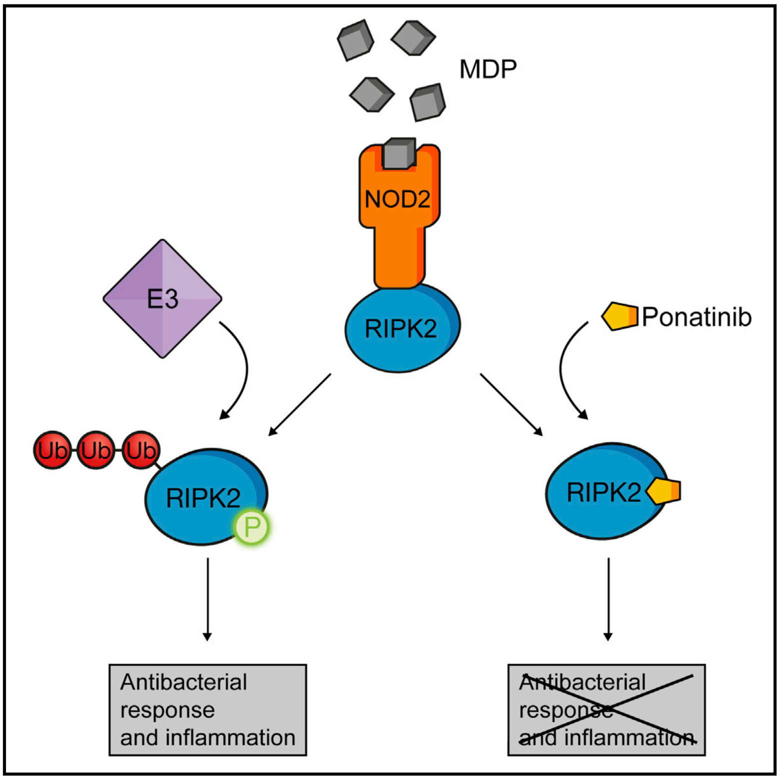

Highlights

- Discovery of type II kinase inhibitors as highly efficacious for RIPK2 inhibition
- RIPK2 autophosphorylation and ubiquitination are blocked by FDA-approved drugs
- RIPK2 crystal structure reveals an allosteric pocket for improving drug selectivity
- NOD-mediated inflammatory signaling is attenuated without affecting TLRs

Authors

Peter Canning, Qui Ruan, Tobias Schwerd, ..., Mads Gyrd-Hansen, Alexei Degterev, Alex N. Bullock

Correspondence

alex.bullock@sgc.ox.ac.uk (A.N.B.),
alexei.degterev@tufts.edu (A.D.)

In Brief

Canning et al. report the structure of the diverse kinase RIPK2 and characterize its inhibition by the FDA-approved drugs ponatinib and regorafenib. The inhibitors prevent the autophosphorylation and ubiquitination of RIPK2 upon NOD2 stimulation, and block downstream NF-κB activation and inflammatory signaling.

Canning et al., 2015, Chemistry & Biology *22*, 1174–1184
September 17, 2015 ©2015 The Authors
http://dx.doi.org/10.1016/j.chembiol.2015.07.017

Chemistry & Biology
Article

Inflammatory Signaling by NOD-RIPK2 Is Inhibited by Clinically Relevant Type II Kinase Inhibitors

Peter Canning,[1] Qui Ruan,[2] Tobias Schwerd,[3] Matous Hrdinka,[4] Jenny L. Maki,[2] Danish Saleh,[5] Chalada Suebsuwong,[6] Soumya Ray,[8] Paul E. Brennan,[1,9] Gregory D. Cuny,[7] Holm H. Uhlig,[3] Mads Gyrd-Hansen,[4] Alexei Degterev,[2,*] and Alex N. Bullock[1,*]
[1]Structural Genomics Consortium, University of Oxford, Old Road Campus, Roosevelt Drive, Oxford OX3 7DQ, UK
[2]Department of Developmental, Molecular & Chemical Biology, Tufts University School of Medicine, 136 Harrison Avenue, Boston, MA 02111, USA
[3]Translational Gastroenterology Unit, Nuffield Department of Medicine and Department of Pediatrics, John Radcliffe Hospital, University of Oxford, Oxford OX3 9DU, UK
[4]Ludwig Institute for Cancer Research, Nuffield Department of Medicine, University of Oxford, Oxford OX3 7DQ, UK
[5]Medical Scientist Training Program and Program in Neuroscience, Sackler School of Graduate Biomedical Sciences, Tufts University School of Medicine, Boston, MA 02111, USA
[6]Department of Chemistry, University of Houston, Houston, TX 77204, USA
[7]Department of Pharmacological and Pharmaceutical Sciences, University of Houston, Houston, TX 77204, USA
[8]Center for Neurologic Diseases, Department of Neurology, Brigham & Women's Hospital and Harvard Medical School, Cambridge, MA 02139, USA
[9]Target Discovery Institute, University of Oxford, NDM Research Building, Roosevelt Drive, Oxford OX3 7LD, UK
*Correspondence: alex.bullock@sgc.ox.ac.uk (A.N.B.), alexei.degterev@tufts.edu (A.D.)
http://dx.doi.org/10.1016/j.chembiol.2015.07.017
This is an open access article under the CC BY license (http://creativecommons.org/licenses/by/4.0/).

SUMMARY

RIPK2 mediates pro-inflammatory signaling from the bacterial sensors NOD1 and NOD2, and is an emerging therapeutic target in autoimmune and inflammatory diseases. We observed that cellular RIPK2 can be potently inhibited by type II inhibitors that displace the kinase activation segment, whereas ATP-competitive type I inhibition was only poorly effective. The most potent RIPK2 inhibitors were the US Food and Drug Administration-approved drugs ponatinib and regorafenib. Their mechanism of action was independent of NOD2 interaction and involved loss of downstream kinase activation as evidenced by lack of RIPK2 autophosphorylation. Notably, these molecules also blocked RIPK2 ubiquitination and, consequently, inflammatory nuclear factor κB signaling. In monocytes, the inhibitors selectively blocked NOD-dependent tumor necrosis factor production without affecting lipopolysaccharide-dependent pathways. We also determined the first crystal structure of RIPK2 bound to ponatinib, and identified an allosteric site for inhibitor development. These results highlight the potential for type II inhibitors to treat indications of RIPK2 activation as well as inflammation-associated cancers.

INTRODUCTION

The nucleotide-binding oligomerization domain-containing proteins NOD1 and NOD2 are cytosolic Nod-like receptor (NLR) family proteins that function in the innate immune system to detect pathogenic bacteria (Philpott et al., 2014). NOD1 is activated upon binding to bacterial peptidoglycan fragments containing diaminopimelic acid (DAP), whereas NOD2 recognizes muramyl dipeptide (MDP) constituents (Chamaillard et al., 2003; Girardin et al., 2003a, 2003b; Inohara et al., 2003). NOD activation induces pro-inflammatory signaling by receptor-interacting protein kinase 2 (RIPK2, also known as RIP2 or RICK), which plays an obligatory and specific role in activation of NOD-dependent, but not Toll-like receptor responses (Park et al., 2007).

Signaling by RIPK2 is dependent on an N-terminal kinase domain with dual Ser/Thr and Tyr kinase activities (Dorsch et al., 2006; Tigno-Aranjuez et al., 2010), as well as a C-terminal caspase activation and recruitment domain (CARD) that mediates CARD-CARD domain assembly with activated NODs (Inohara et al., 1999; Ogura et al., 2001b). Once engaged, RIPK2 is activated by autophosphorylation (Dorsch et al., 2006) and further targeted by XIAP (X-linked inhibitor of apoptosis) and other E3 ligases for non-degradative polyubiquitination (Bertrand et al., 2011; Damgaard et al., 2012; Tao et al., 2009; Tigno-Aranjuez et al., 2013; Yang et al., 2007, 2013). The ubiquitin-conjugated protein subsequently activates the TAK1 and IKK kinases, leading to upregulation of both the mitogen-activated protein kinase and nuclear factor κB (NF-κB) signaling pathways (Kim et al., 2008; Park et al., 2007). In addition, RIPK2 induces an antibacterial autophagic response by signaling between NODs and the autophagy factor ATG16L1 (Cooney et al., 2010; Homer et al., 2012).

The NOD2-RIPK2 pathway has attracted special interest due to the role of this signaling node in granulomatous inflammatory diseases, including inflammatory bowel disease (IBD). Such pathologies can arise from either positive or negative dysregulation of the pathway (Caruso et al., 2014; Jostins et al., 2012; Philpott et al., 2014). Genetic variants in *NOD2* are the strongest susceptibility factor to Crohn's disease (Hugot et al., 2001; Jostins et al.,

2012; Ogura et al., 2001a). Crohn's disease-associated mutations that abrogate NOD2 binding to MDP may induce excessive inflammatory signaling from other pattern recognition receptors, including NOD1 (Couturier-Maillard et al., 2013; Inohara et al., 2003). In contrast, mutations in the second major Crohn's disease susceptibility factor, ATG16L1, disrupt an inhibitory interaction with NOD2 and consequently increase the activation of RIPK2 (Sorbara et al., 2013). Excessive RIPK2 activation has also been reported in pediatric Crohn's disease (Negroni et al., 2009). In addition, gain of function in the NOD2-RIPK2 pathway has been linked to Blau syndrome, early-onset sarcoidosis, allergic airway inflammation, and multiple sclerosis (Goh et al., 2013; Jun et al., 2013; Shaw et al., 2011). Overall, these data establish RIPK2 as a key molecule for the understanding of IBD pathogenesis as well as a potential therapeutic target in a wide spectrum of inflammatory and autoimmune diseases.

Importantly, the kinase activity of RIPK2 is essential for its stability and function, offering a promising rationale for small-molecule intervention (Nembrini et al., 2009; Tigno-Aranjuez et al., 2010). To date, all studies of RIPK2 have focused on small molecules of the type I inhibitor class, which bind to the kinase ATP pocket and are ATP competitive. This approach was first validated using SB203580, a pyridinyl imidazole inhibitor of p38, which showed additional inhibition of RIPK2 in vitro and was efficacious in a Crohn's disease model in mice (Argast et al., 2005; Hollenbach et al., 2005). Further proof of concept was subsequently attained using the clinical epidermal growth factor receptor inhibitor, gefitinib, which also inhibited RIPK2 and improved disease burden in a spontaneous model of Crohn's disease-like ileitis (Tigno-Aranjuez et al., 2010, 2014). Finally, a new class of macrocyclic RIPK2 inhibitors has recently been described as capable of inhibiting cellular NOD-dependent inflammatory responses at 200–500 nM (Tigno-Aranjuez et al., 2014). These molecules also displayed promising in vivo activity in models of Crohn's ileitis as well as NOD-driven peritonitis (Tigno-Aranjuez et al., 2014).

Here, we show that the inhibition of RIPK2 signaling can be improved by two orders of magnitude by using type II inhibitors that alternatively target the inactive "DFG-out" conformation of the kinase domain, including the US Food and Drug Administration (FDA)-approved drugs ponatinib, sorafenib, and regorafenib. Type II binding is confirmed by the first crystal structure of RIPK2 solved in complex with ponatinib, which reveals an allosteric site suitable for the rational design of RIPK2-selective small molecules. The identified clinical inhibitors disrupt RIPK2 activation in monocytes and macrophages to selectively reduce inflammatory signaling from NOD1 and NOD2, but not tumor necrosis factor (TNF) induction from Toll-like receptors. Overall, this work identifies the structural basis to investigate the therapeutic potential of RIPK2 inhibition in inflammatory diseases by defining clinically relevant scaffolds for the development of selective RIPK2 inhibitors.

RESULTS

Identification of Ponatinib as a Potent Inhibitor of RIPK2

To identify inhibitors of human RIPK2, we purified the recombinant kinase domain from Sf9 insect cells and screened it against a library of small-molecule kinase inhibitors using a fluorescence-based thermal shift assay (Niesen et al., 2007). In this assay, the previously reported type I inhibitors gefitinib and LDN-193189 yielded large thermal shift (ΔT_m) values of 9.5°C and 12.1°C, consistent with their respective IC_{50} values of 49 nM (Tigno-Aranjuez et al., 2014) and 25 nM (Vogt et al., 2011). By comparison, the type II inhibitor ponatinib yielded a remarkable ΔT_m of 23.1°C and was identified as the most potent hit (Table S1). To further explore type II inhibitors as putative preferred scaffolds for RIPK2, we set out to solve the co-crystal structure of RIPK2 with ponatinib. Protein crystallization was hindered initially by heterogeneous phosphorylation, but was enabled following treatment with lambda phosphatase. Viable crystals were grown in space group $P2_12_12_1$ with two molecules in the asymmetric unit. The structure was solved by molecular replacement and refined at 2.75 Å resolution. Crystallographic data collection and refinement statistics are presented in Table S2.

Structural Features of RIPK2

The structure of RIPK2 exhibits the canonical bilobal kinase fold followed by a 16-residue αJ helix that packs alongside the loop connecting the αD and αE helices (Figure 1A). This C-terminal structural element is common in STE family kinases, but is present additionally in RIPK1-3 (Figure 1B). The bound ponatinib inhibitor occupies the ATP pocket established between the N- and C-terminal lobes of the kinase. As a result, RIPK2 displays an inactive conformation of the catalytic domain characterized by a "DFG-Asp out, αC-Glu in" configuration. The β3 lysine (Lys47) and αC glutamine (Glu66) establish the catalytically relevant salt bridge, whereas the DFG aspartate (Asp164) is flipped away from the active site, rendering the enzyme inactive. Of note, the activation segment helix found in the crystal structures of the two homologous kinases, RIPK1 and RIPK3, is not present in RIPK2, although a significant portion of the activation loop was not visible in the electron density map and not modeled (Figure 1B). Residues from this helix, in particular Ser161, are known to contribute to the binding of RIPK1 to selective small-molecule inhibitors, necrostatins (Xie et al., 2013a). Interestingly, RIPK2 also contains several unusual sequence changes in its catalytic motifs that are not conserved in other RIPKs. The typical HRD triad in the catalytic loop is changed to HHD, while the activation loop APE motif is changed to PPE (Figure 1C). Moreover, the kinase domain of RIPK2 as a whole displays only 33% sequence identity with other proteins in the PDB (namely RIPK1 and RIPK3), indicating its broader diversity.

Oligomerization into multi-protein signaling complexes is a key part of the activation mechanism in the RIPK family. The RIPK2 structure reveals a homodimeric packing arrangement similar to that of RIPK3 (Xie et al., 2013b) and consistent with the dimeric state observed in solution by analytical gel filtration (Figure S1). The protein interface is highly symmetrical, with the two active sites facing in opposite directions and rotated approximately 90° relative to one another (Figure 2). Binding is supported by the αJ helices, which pack against each other in an antiparallel fashion, and form both hydrophobic interactions and a symmetrical pattern of hydrogen bonding between the side chains of Lys310 and Glu299 and the side chains of His159 and Glu157 (Figure 2). Additional contacts are made between the β2-β3

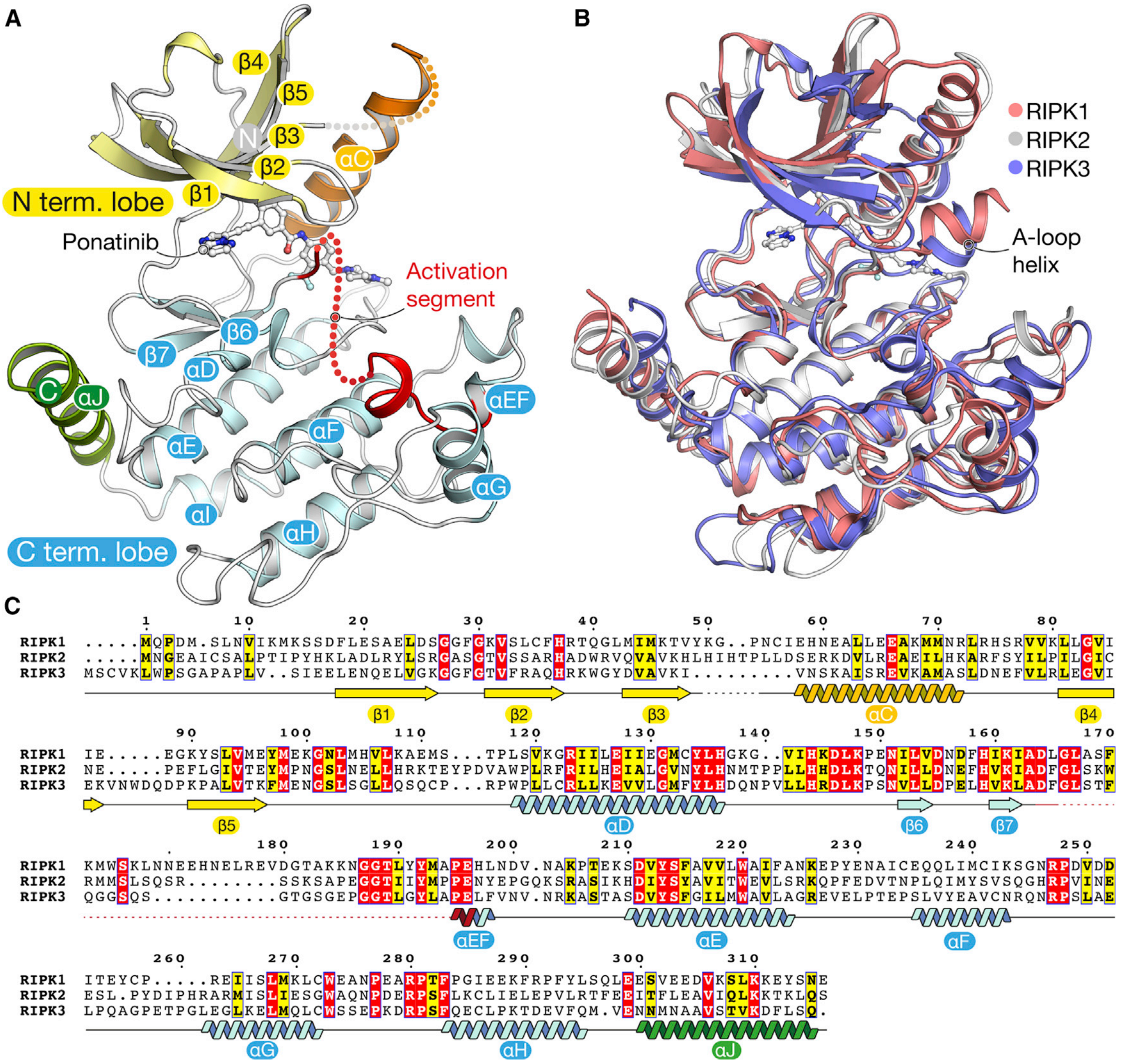

Figure 1. Structural Features of the RIPK2 Kinase Domain

(A) Crystal structure of the kinase domain of human RIPK2 showing the bound ponatinib molecule. See also Tables S1 and S2.

(B) Superposition of the kinase domains of RIPK1 (pink, PDB: 4NEU), RIPK2 (white), and RIPK3 (blue, PDB: 4M69). The activation segment helix present in the structures of RIPK1 and RIPK3 is marked.

(C) Sequence alignment of the kinase domains of human RIPK1–3. Residue numbers refer to the RIPK2 sequence, and secondary structure elements labeled in (A) are marked.

loop of one subunit and the αE and αI helices of the other (Figure 2).

The Binding Mode of Ponatinib and Other Clinically Relevant Type II Inhibitors

Ponatinib (Figure 3A) has been crystallized previously only in complex with tyrosine kinases. The binding mode in RIPK2 resembles that of DDR1 and KIT with an intact conformation of the β1-β2 hairpin, rather than the collapsed loop of Abl (Figure 3B). The imidazo[1,2-*b*]pyridazine head group establishes a single hydrogen bond to the hinge residue Met98 as well as hydrophobic interactions with Tyr97 and Leu24. The central linker forms two additional hydrogen bonds with the side chain of Glu66 and the main-chain nitrogen of Asp164. The trifluoromethyl group occupies the hydrophobic pocket vacated by the inverted DFG motif, while the protonated methylpiperazine forms an ionic-dipole interaction with the main-chain oxygen atoms of Leu143 and His144, positioned within the αD-β6 loop (Figure 3B). Importantly, the structure also reveals opportunities for the development of RIPK2-selective molecules. In particular, the allosteric hydrophobic pocket occupied by the trifluoromethyl group is greatly enlarged in RIPK2 due to the presence of Ala73 in the αC helix (Figure 3C). Nearly all kinases contain a bulky side chain at this position, such as Leu70 in RIPK1. Thus, larger chemical groups at this site will not only increase potency for RIPK2, but will also sterically restrict inhibitor binding to the wider kinome.

Regorafenib and sorafenib are two other multi-targeted clinical type II inhibitors that share a phenyl-urea-trifluoromethylphenyl core (Figure 3A). Docking studies suggested that these

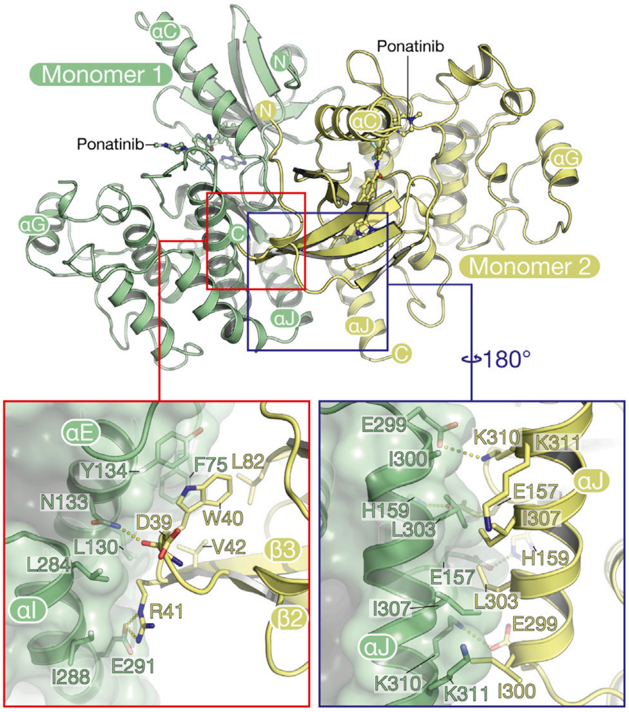

Figure 2. The RIPK2 Kinase Domain Is Dimeric

The main panel shows the overall arrangement of the two monomers, and the inset panels show selected residues in the dimer interface. See also Figure S1.

molecules can assume a binding pose similar to that of ponatinib in RIPK2, including a total of five hydrogen bonds formed with Met98, Glu66, and Asp164 (Figures 3D and S2). To test these predictions, we performed in vitro kinase assays using the ADP-Glo assay. Ponatinib inhibited the activity of recombinant RIPK2 with an IC_{50} value of 6.7 nM, demonstrating inhibition comparable with that of Abl (IC_{50} = 1.6 nM, Figure 3E). By comparison, the inhibition of RIPK2 by regorafenib and sorafenib was an order of magnitude weaker (IC_{50} values of 41 and 75 nM, respectively), but similar to the activity of the previously reported type I inhibitor gefitinib (IC_{50} = 51 nM, Figure 3F). This likely reflects the loss of the ionic-dipole interactions formed by the distal piperazine of ponatinib with the main-chain oxygen atoms of Leu143 and His144. Consistently, changes to this moiety, which is present in ponatinib but not sorafenib or regorafenib, were reported to partially attenuate inhibition of Abl kinase (Huang et al., 2010).

Inhibition of Cellular RIPK2 Activation

The kinase activity of RIPK2 has been shown to mediate the activation of inflammatory signaling by the NOD1/2 family of peptidoglycan receptors. Autophosphorylation of Ser176 in the kinase activation segment has been identified as a specific marker of RIPK2 activation (Dorsch et al., 2006; Nachbur et al., 2015). To confirm inhibition of cellular RIPK2 activation by the inhibitors, we therefore analyzed changes in phospho-Ser176-RIPK2 (p-RIPK2) following stimulation of NOD2-expressing HEK293 cells with L18-MDP ligand (a lipidated form of MDP with enhanced potency). L18-MDP caused a rapid increase in endogenous p-RIPK2 that was inhibited by low nanomolar concentrations of ponatinib, sorafenib, and regorafenib (Figure 4A). Ponatinib displayed the highest activity, completely blocking RIPK2 phosphorylation at 10 nM, followed by regorafenib and sorafenib. This was consistent with the order of activities observed in the in vitro kinase assays. Furthermore, by blocking RIPK2 activation the inhibitors prevented the phosphorylation and subsequent degradation of IκBα (Figures 4A and S3), which is required for activation of NF-κB and induction of inflammatory gene expression (Cramer and Muller, 1999). Surprisingly, the type I inhibitor gefitinib showed much lower activity in cells relative to the in vitro assays (Figure 4A). This may partly reflect its ATP-competitive mode of action, with potential loss of activity due to the high concentrations of ATP in cells. None of the molecules significantly inhibited phosphorylation of ERK1/2 or global levels of phosphotyrosine proteins, which served as negative controls (Figure 4A). In addition, none of the inhibitors affected the viability or morphological appearance of HEKBlue or RAW264.7 macrophage cells, except for 100 nM ponatinib, which caused rounding of RAW cells and likely reflects off-target activity at the highest concentration (Figure S4).

To further quantify cellular RIPK2 inhibition, we measured the downstream activation of NF-κB in the same HEKBlue cells, which were also stably transfected with an NF-κB-SEAP reporter. Ponatinib again displayed the most potent, low nanomolar activity (EC_{50} = 0.8 nM), followed by regorafenib and sorafenib (Figure 4B). By comparison, gefitinib inhibited NF-κB activity with an EC_{50} value of 7.8 μM, consistent with the p-RIPK2 data. Overall, these data identified ponatinib, sorafenib, and regorafenib as a new class of low nanomolar inhibitors of RIPK2 activation in cells.

Since ponatinib is a potent inhibitor of Abl, we further examined whether Abl may contribute to the inhibition of the NOD2 responses in HEK cells in addition to RIPK2. We found that a different type II inhibitor of Abl, nilotinib, did not inhibit either RIPK2 in vitro (Figure S5A) or the NOD2 response in HEKBlue cells (Figure S5B). Because ponatinib is a large lipophilic molecule, which may cause non-specific effects, we also sought to identify close analogs that would lack activity against RIPK2. We noticed that methyl of the central phenyl ring of ponatinib inserts into the shallow lipophilic pocket formed by aliphatic side chains of Val32, Lys47, Ile93, and Thr95. A bulkier *tert*-butyl side chain (CS6, Figure 3A) was designed based on this hypothesis and synthesized using previously reported methods (Huang et al., 2010; Najjar et al., 2015). Introduction of the *tert*-butyl led to the loss of inhibition of both RIPK2 and Abl kinase activity (Figure S5A), providing us with a bulky and lipophilic control molecule. Importantly, CS6 did not inhibit the MDP response in HEKBlue cells (Figure S5B). These data and the lack of cellular activity of nilotinib further confirmed the specific role of RIPK2 inhibition in these assays.

To explore the mechanism of RIPK2 inhibition, we tested the effects of ponatinib on the required interaction with NOD2. For this, we used U2OS cells that inducibly expressed HA-tagged NOD2 and performed HA-immunoprecipitation to recover bound RIPK2. As expected, HA-NOD2 expression was detected only in the presence of doxycycline, and induced

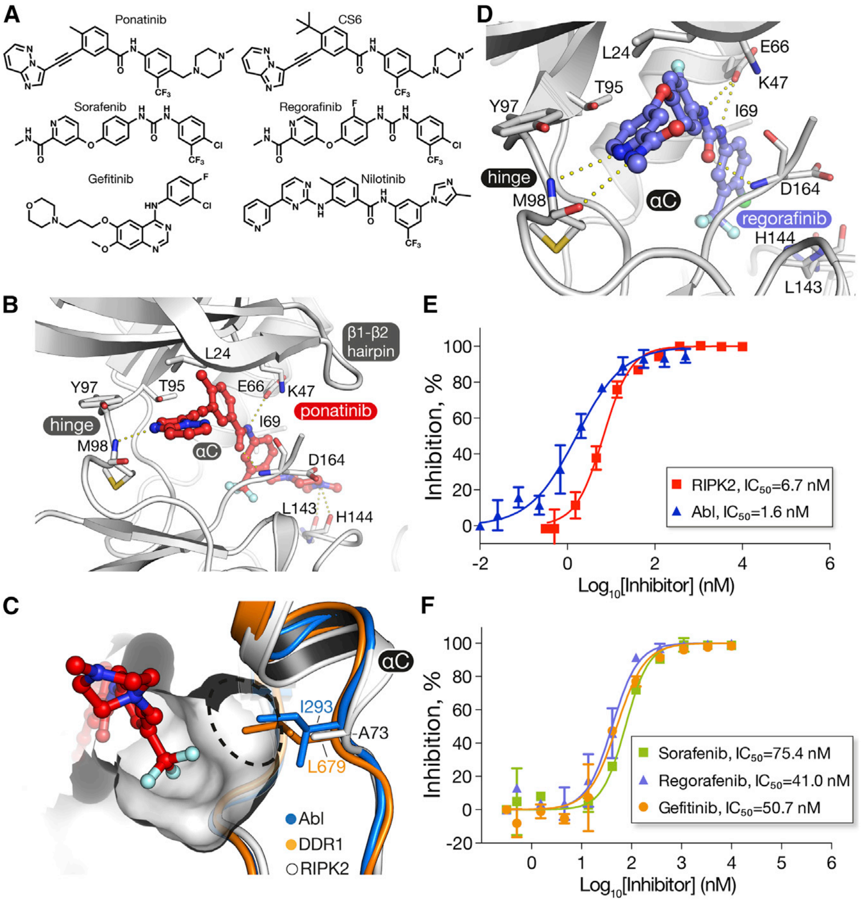

Figure 3. Inhibition of Abl and RIPK2 Kinases In Vitro

(A) Chemical structures of inhibitors used in this study.

(B) Binding mode of ponatinib to RIPK2.

(C) The DFG-out hydrophobic pocket in RIPK2 is almost uniquely large due to the small Ala73, which replaces the Ile/Leu residues of Abl/DDR2. A dashed line highlights the expanded pocket area in RIPK2, which may accommodate larger substitutions of the trifluoromethyl group for improved selectivity.

(D) Predicted binding mode of regorafenib. Docking was performed with ICM-Pro (Molsoft). See also Figure S2.

(E) Dose-response curves showing ponatinib inhibition of RIPK2 and Abl.

(F) Dose-response curves for RIPK2 inhibition by sorefanib, regorafenib, and gefitinib. In (E) and (F), experiments were performed in duplicate; error bars indicate SD values. Kinase activity was measured using the ADPGlo assay. Non-linear curve fitting to calculate IC_{50} values was performed using Prism software. See also Figure S5.

a robust interaction with RIPK2 (Figure 4C) as well as downstream NF-κB activation (Figure 4D). Interestingly, the NOD2-RIPK2 interaction was stably maintained in the presence of 100 nM ponatinib (Figure 4C), whereas NF-κB activation was completely disabled (Figure 4D). These results indicated that ponatinib acts to inhibit the activation of the kinase domain of RIPK2 but does not interfere with the C-terminal CARD domain, which recognizes NOD2.

Ponatinib Potently Abrogates RIPK2 Ubiquitination and Induction of Inflammatory Cytokines

To examine inhibition of RIPK2-induced inflammation in a more physiological context, we analyzed the receptor signaling pathway in human monocytic THP-1 cells and in mouse macrophage RAW264.7 cells in response to different PRR (pattern recognition receptor) ligands and cytokines. Stimulation of NOD2 by MDP leads to rapid ubiquitination of RIPK2 by XIAP and other ubiquitin ligases, a process required for downstream signaling and transcription of NF-κB target genes (Damgaard et al., 2012, 2013; Fiil et al., 2013; Yang et al., 2013). Consistent with the relative potency of the kinase inhibitors against RIPK2 activity, pre-treatment of THP-1 cells with 100 nM ponatinib completely blocked L18-MDP-induced RIPK2 ubiquitination, whereas regorafenib and gefitinib at the same concentration had less robust inhibitory effects (Figures 5A and S6). Ponatinib also blocked the downstream degradation of IκBα, whereas the other inhibitors had little or no detectable effect (Figure 5A). Importantly, ponatinib interfered with RIPK2 ubiquitination in a dose-dependent manner. Concentrations as low as 5–10 nM reduced the extent and length of ubiquitin-modified RIPK2, while RIPK2 ubiquitination was completely blocked at concentrations of 25 nM or higher (Figure 5B). By contrast, ponatinib had no obvious effects on ubiquitination of the related kinase RIPK1 or IκBα degradation following treatment of THP-1 cells with TNF (Figure 5C).

We next analyzed the pattern of inflammatory gene expression in RAW264.7 macrophage cells. Consistent with the results obtained in THP-1 cells, MDP stimulation led to robust increases in *CCL4*, *CXCL2*, and *RANTES* mRNA levels that were efficiently inhibited by low nanomolar (1–10 nM) concentrations of ponatinib (Figure 6A). Regorafenib was similarly active at 10–100 nM while sorafenib was slightly less effective (Figure 6A). Comparable results were observed following RIPK2 stimulation with the NOD1 agonist, Tri-DAP (Figure 6B). In contrast, ponatinib and the other inhibitors did not block the mRNA induction by a different class of PAMPs (pathogen-associated molecular patterns), such as agonists of Toll-like receptors 2 and 4 (Pam3CSK4 and lipopolysaccharide [LPS], respectively) (Figures 6C and 6D). The only exception was 100 nM ponatinib, which partially attenuated both the LPS and Pam3CSK4 responses, likely reflecting nonspecific activity of this inhibitor at higher concentrations. Overall, we were able to observe selective and efficient inhibition of RIPK2-dependent NOD1/2 responses by both ponatinib and regorafenib at low nanomolar concentrations and by sorafenib at ≤100 nM.

Modulation of NOD1/2-RIPK2-XIAP-mediated signaling has been proposed as a therapeutic approach for inflammatory disorders (Jun et al., 2013). Therefore, we investigated whether

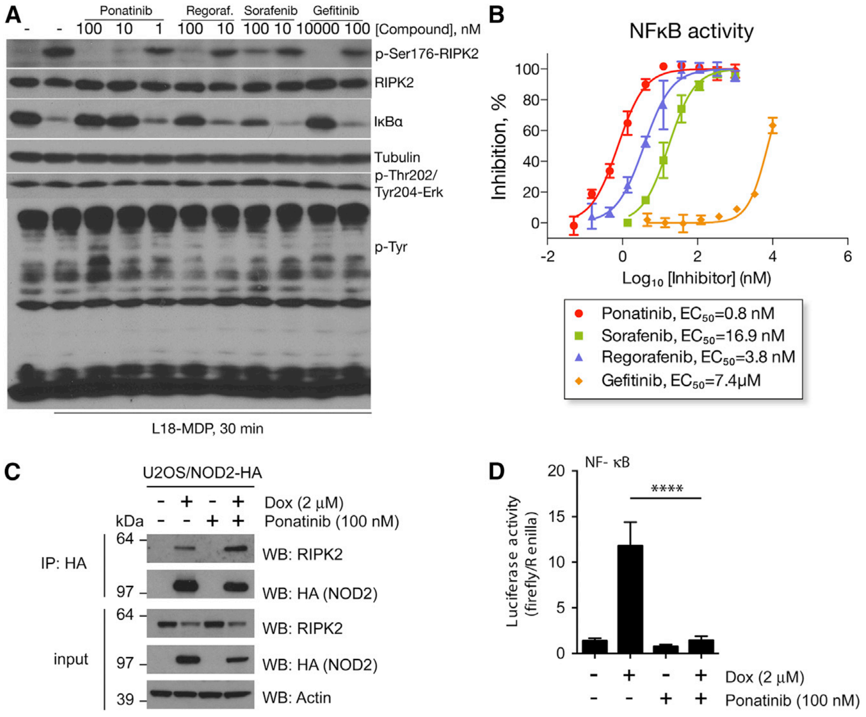

Figure 4. Inhibition of RIPK2 Activation in HEKBlue and U2OS Cells

(A) Phosphorylation changes in HEKBlue cells. Cells were treated with indicated concentrations of inhibitors, followed 30 min later by stimulation with 1 μg/ml L18-MDP. Cells were harvested after 30 min, and changes in protein phosphorylation were analyzed by western blotting. Levels of tubulin and total RIPK2 were used as loading controls.

(B) Inhibition of NF-κB activation in HEKBlue cells. HEKBlue reporter cells, expressing NOD2 and NF-κB-SEAP reporter, were treated with 6–8 concentrations of each inhibitor in triplicate followed by stimulation with 1 μg/ml L18-MDP for 8 hr. SEAP activity was detected using HEKBlue media with detection of absorbance at 620 nM in a Wallac3V plate reader. Non-linear curve fitting to calculate EC_{50} values was performed using Prism software. Experiments were performed in triplicate, error bars indicate SD values. See also Figure S5.

(C) Interaction of RIPK2 with inducibly overexpressed HA-NOD2 in U2OS cells in the presence of ponatinib. The immunoprecipitation was performed using anti-HA agarose after 24 hr of HA-NOD2 induction and ponatinib treatment. Dox, doxycycline. Representative result of the experiment performed three times.

(D) NF-κB dual luciferase reporter assay in U2OS cells inducibly overexpressing HA-NOD2 and treated with ponatinib for 24 hr. Dox, doxycycline. Experiment performed three times in three technical replicates. Error bars represent ±SEM. ****$p < 0.0001$.

NOD2 receptor activation in primary human monocytes can be inhibited to an extent similar to that observed for the described epithelial and leukocyte cell lines. Ponatinib, regorafenib, and gefitinib inhibited the production of TNF in peripheral blood-derived monocytes after L18-MDP stimulation (Figure 7A). The most potent inhibition was observed with ponatinib, which completely abrogated NOD2-dependent TNF production at low nanomolar concentrations down to 10 nM (Figure 7B). Regorafenib was similarly effective at 100 nM, whereas a much higher concentration of gefitinib (10 μM) was required for complete inhibition (Figure 7B). TNF production by LPS stimulation was not affected at these inhibitor concentrations (Figures 7A and 7B), and was attenuated only by ponatinib at the much higher concentration of 1 μM (Figure 7B). Similar inhibitor efficacies were observed in the human monocytic cell line THP-1, although baseline response in these cells was low and 10 μM gefitinib also inhibited the LPS pathway (Figure S7).

Overall, the results on primary human monocytes are fully consistent with the earlier data obtained in laboratory cell lines, suggesting that the tested cell lines provide an accurate tool to assess the potency of RIPK2 inhibition. Furthermore, they support the hypothesis that tyrosine kinase inhibitors can be used in primary human cells to selectively target RIPK2.

DISCUSSION

To date, all reported RIPK2 inhibitors have been ATP-competitive type I molecules, such as the clinical drug gefitinib. Importantly, we observed that the cellular activity of this type I inhibitor was vastly outperformed by the identified type II inhibitors, despite their comparable potencies in the in vitro kinase assay (e.g., comparing regorafenib and gefitinib). Multiple factors could contribute to these differences. For example, the endogenous full-length RIPK2 may display a higher affinity for ATP than the isolated kinase domain, resulting in greater competition for type I binders. Alternatively, the DFG-out conformation might represent a preferred conformation in cells, or perhaps form a dominant-negative species. Indeed, activation loop phosphorylation in JAK2 is incompatible with the binding of type II inhibitors, but permissible with inhibitors of the type I class (Andraos et al., 2012). While the mechanistic basis for these differences remains to be further elucidated, these data clearly identify type II inhibitors as the most efficacious molecular class for targeting RIPK2.

The potency of the type II inhibitors also affords new tools with which to investigate the molecular mechanisms of RIPK2 signaling and the effects of kinase inhibition. We found that ponatinib blocked the activation of the kinase domain of RIPK2 without affecting the C-terminal CARD domain and its engagement of NOD2. A similar breakdown in RIPK2 activation has been reported for a NOD1 variant containing the rare Asn43Ser polymorphism (Mayle et al., 2014). However, in the absence of structural information there is little understanding of how the CARD domain status of RIPK2 is communicated to the kinase domain. Potentially, new stimulatory interactions could be formed or inhibitory interactions broken. All the tested inhibitors were able to block the phosphorylation of the kinase activation loop (Ser176), which is a known marker for RIPK2 activation. Activated RIPK2 is also targeted by multiple E3 ligases for polyubiquitination by mechanisms that are

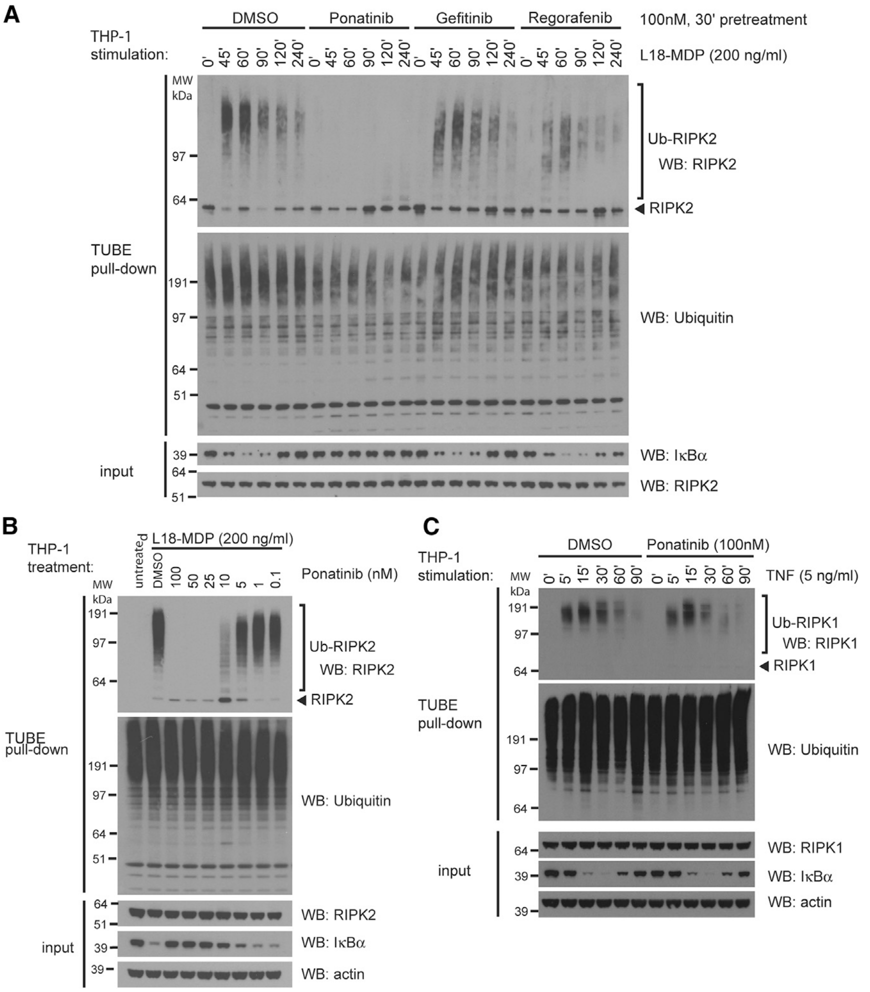

Figure 5. Inhibition of NOD2-Dependent Ubiquitination and Signaling

(A–C) THP-1 cells were pre-treated with kinase inhibitors or DMSO for 30 min and stimulated with 200 ng/ml L18-MDP (A, B) or TNF (C) as indicated. At the indicated time points, cells were lysed and ubiquitinated proteins were isolated using TUBE reagent. The isolated ubiquitinated proteins and input material were analyzed by immunoblotting. See also Figure S6.

yet to be structurally characterized. Unexpectedly, we found that ponatinib could inhibit this modification completely, whereas type I inhibitors have previously only caused a delay in ubiquitination (Nachbur et al., 2015). This result suggests that RIPK2 binding to E3 ligases is more strictly regulated than previously imagined. Thus, it will be interesting to decipher the precise mechanism of E3 recruitment in future work. We also observed excellent selectivity of the inhibitors toward MDP-dependent signaling relative to LPS-dependent pathways. This result is noteworthy given the known promiscuity of ponatinib within the kinome (Zhao et al., 2014). It also further supports the specificity of RIPK2 for NOD-dependent signaling (Park et al., 2007).

In a separate study, we have found that ponatinib is also an efficient inhibitor of RIPK1 and RIPK3 kinase activity in necroptosis (Najjar et al., 2015), making this molecule the only known pan-RIPK inhibitor. The current work using ponatinib and TNF stimulation, in line with previous reports, shows that RIPK1 kinase activity is dispensable for its ubiquitination as well as for the downstream degradation of IκBα (Lee et al., 2004). Thus, the experiments with ponatinib reveal important differences in the mechanisms of RIPK1 and RIPK2. Nonetheless, the ability of ponatinib to simultaneously target multiple RIPKs may be of interest for investigating inflammatory disorders. By contrast, inhibition of RIPK1/RIPK3 was not observed with sorafenib or regorafenib (data not shown). These two inhibitors lack the methylpiperazine group that is present in ponatinib. The activation segments in RIPK1/RIPK3 also show a high propensity to form a short α helix that is not observed in the RIPK2 structure. Additional co-structures and SAR may be required to understand the selectivity differences that must exist between the DFG-out (DLG in RIPK1) pockets of these kinases.

Importantly, ponatinib, regorafenib, and sorafenib are FDA-approved medications that are used clinically against various forms of cancer. Inhibition of RIPK2 represents a novel off-target activity, although microbiota-driven inflammation has emerged as a potentially important player in tumorigenesis (Elinav et al., 2013; Saxena and Yeretssian, 2014). IBD is also a known risk factor for colorectal cancer (Sebastian et al., 2014). Nonetheless, the broad kinase selectivity of these drugs currently prohibits their use in chronic inflammatory conditions. In particular, ponatinib can cause serious adverse events including vascular thrombosis (Cortes et al., 2013). Sorafenib and regorafenib are better tolerated and are used clinically at low-micromolar doses that are higher than those used in our study (Moore et al., 2005; Mross et al., 2012). These molecules are therefore potentially interesting tools with which to further explore RIPK2 function in pre-clinical models of colitis and other inflammatory conditions.

Finally, this work also suggests that more selective compounds may be derived by targeting the expanded allosteric pocket identified in the RIPK2 structure. For example, the trifluoromethyl group could be replaced with a larger substitute, such as methoxymethyl, isopropyl, or isopropoxy. All of these groups have the potential to extend further into the RIPK2 pocket without dramatic changes in hydrogen bonding or lipophilicity. In addition, changes to the hinge-binding "head" group could be considered by comparison with RIPK2-selective type I inhibitors such as WEHI-345 (Nachbur et al., 2015). The recently identified macrocycles OD36 and OD38 would, however, form steric clashes in a type II binding mode (Tigno-Aranjuez et al., 2014). The toolbox of new compounds will form valuable reagents for

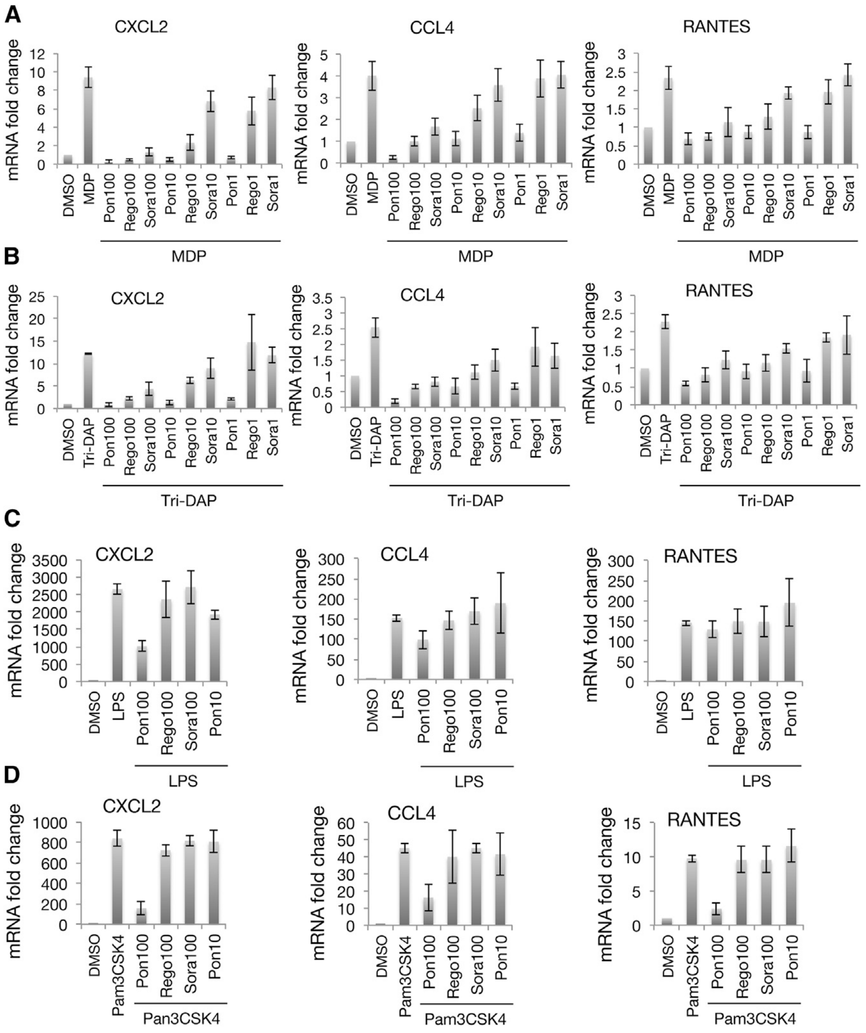

Figure 6. Inhibition of NOD-Dependent Inflammatory Gene Expression in RAW264.7 Cells

(A and B) Cells were pre-treated with 1, 10, or 100 nM inhibitors for 30 min and stimulated with 10 μg/ml MDP (A) or Tri-DAP (B) for 18–24 hr in triplicate. RNA samples were isolated and changes in gene expression were analyzed using gene-specific primers using SYBR qRT-PCR. All values were normalized to the levels of *GAPDH*.

(C and D) Lack of inhibition of Toll-like receptor-dependent inflammatory gene expression in RAW264.7 cells. Experiments were performed as described above, except cells were stimulated with 10 ng/ml *E. coli* LPS (C) or 500 ng/ml Pam3CSK4 (D).

All experiments were performed in triplicate; error bars indicate SD values.

further investigation of the complexity of RIPK2 regulation in both normal signaling and pathobiology.

SIGNIFICANCE

Clinical kinase inhibitors have been utilized almost exclusively in oncology. The recent approval of JAK inhibitors for the treatment of inflammatory conditions, in particular rheumatoid arthritis, has demonstrated the potential of this drug class to target other indications. RIPK2 is one emerging therapeutic target in inflammation strongly supported by genetic evidence of activating NOD2 mutations in the monogenic autoinflammatory disease Blau syndrome, characterized by early-onset granulomatous arthritis, uveitis, and dermatitis. To date, pre-clinical validation studies for inflammatory conditions have largely focused on the clinical inhibitor gefitinib, which binds to kinases in their active conformation. Here, we show that RIPK2 is highly amenable to type II inhibition, which affords dramatic improvements in cellular potency. Furthermore, the most potent molecules, ponatinib, regorafenib, and sorafenib, extend the available inhibitor activities from micromolar to subnanomolar, allowing fine-tuning insights into RIPK2 regulation. In particular, binding of ponatinib to the kinase domain is sufficient to block all ubiquitination on RIPK2, and demonstrates the requirement for this modification for the downstream destruction of IκBα, in contrast to the requirements of RIPK1. In addition, regorafenib offers selectivity for RIPK2 over RIPK1/3 as well as low nanomolar potency to alleviate some "off-target" effects. Further scaffold improvements to overcome such liabilities are also suggested by the presented RIPK2 structure. Overall, this work identifies advanced tools to investigate the functional role of RIPK2 in control of the intestinal microbiota as well as clinically relevant scaffolds to explore the therapeutic potential of RIPK2 inhibition in inflammatory diseases.

EXPERIMENTAL PROCEDURES

Cells and Reagents

Detailed information on reagents, qPCR, and immunoblotting is provided in the Supplemental Experimental Procedures.

Purification of RIPK2

Human RIPK2 (Uniprot: O43353, residues 8–317) was expressed in Sf9 insect cells, and purified by nickel affinity and size-exclusion chromatography. Detailed information is provided in the Supplemental Experimental Procedures.

Crystallization and Structure Determination

RIPK2 was concentrated to 3.7 mg/ml. Crystals with ponatinib were grown in sitting drops using a reservoir solution containing 0.1 M ammonium citrate and 16% (w/v) polyethylene glycol 3350. Diffraction data were collected on Diamond Light Source beamline I04. Detailed information on structure determination is provided in the Supplemental Experimental Procedures.

Thermal Shift Assay

RIPK2 protein at 2 μM concentration was mixed with inhibitor compounds at 10 μM and a 1:1,000 dilution of SyproOrange fluorescent dye (Invitrogen).

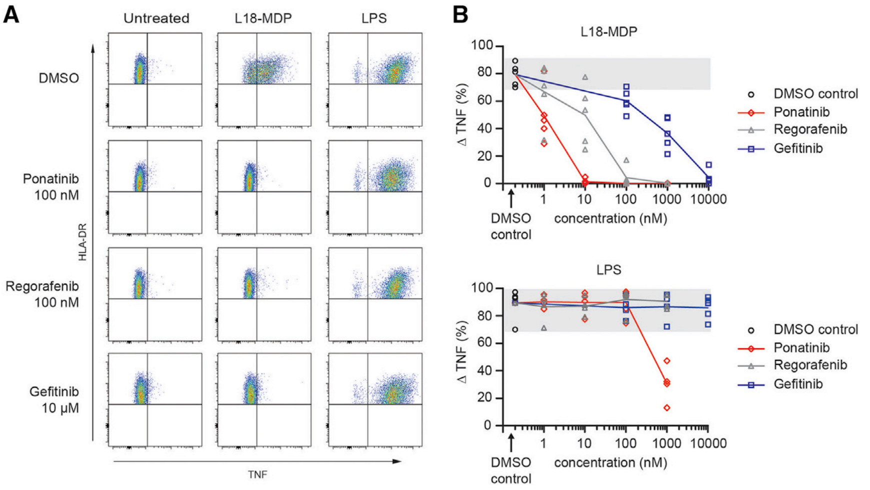

Figure 7. Dose-Dependent Inhibitory Effect of Ponatinib, Regorafenib, and Gefitinib on MDP-Induced TNF in Primary Human Monocytes

Intracellular TNF production was determined by flow cytometry in rested monocytes of healthy blood donors cultured in the presence or absence of L18-MDP (200 ng/ml) or LPS (200 ng/ml). Cells were pre-treated with the indicated concentrations of inhibitors for 60 min before receptor activation.
(A) Representative fluorescence-activated cell sorting density blots of TNF-positive monocytes among all single, live, HLA-DR$^+$, and CD14$^+$ cells.
(B) Induction of TNF in monocytes after L18-MDP or LPS stimulation is calculated as ΔTNF, subtracting the frequency of TNF-producing monocytes cultured in medium alone from the percentage of TNF-positive monocytes following activation. Experimental conditions are measured in 4–5 healthy donors. Individual replicates and the mean connected by a line are shown. Gray background indicates range without inhibitors.
See also Figure S7.

Fluorescence-based thermal shift assays were performed in an Mx3005p real-time PCR machine (Agilent) as described by Niesen et al. (2007).

ADPGlo In Vitro Kinase Assays

For ADPGlo (Promega) assays, 1 ng of Abl or 10 ng of RIPK2 was diluted in reaction buffer (40 mM Tris-HCl [pH 7.5], 20 mM $MgCl_2$, 0.5 mM DTT, and 0.01% BSA) supplemented with 50 μM ATP and a 10-point dose range of inhibitors. Detailed information is provided in the Supplemental Experimental Procedures.

HEKBlue Activation Assay

HEKBlue cells (1×10^5 cells/ml) were resuspended in QUIATI-Blue detection medium and seeded into 96-well plates (100 μl/well). Cells were treated with small-molecule inhibitors and 1 μg/ml L18-MDP for 8–10 hr. Absorbance at 620 nM was determined at the end of the incubation using a Victor3V plate reader (PerkinElmer). Values of empty media were subtracted from all experimental samples. Resulting specific signal values were used to calculate inhibition: % = (1 − [control (DMSO, L18-MDP) − sample (compound, L18-MDP)]/[control (DMSO, L18-MDP) − control (DMSO)]) × 100. EC_{50} values were determined using non-linear regression in the Prism software package (GraphPad).

Purification of Ubiquitin Conjugates

The ubiquitin conjugates were purified using GST-Tandem Ubiquitin Binding Entities (TUBE; Fiil et al., 2013). In brief, treated THP-1 cells ($6–10 \times 10^6$) were washed with PBS and lysed in 400 μl of ice-cold lysis buffer containing TUBE on ice for 30 min. Cleared lysates were incubated with Glutathione Sepharose 4B resin (Amersham) with agitation at 4°C overnight. The beads were washed four times with PBS-Tween (0.1%) and the bound proteins were released by heating the beads in reducing SDS sample buffer. The samples were resolved on pre-cast gradient gels (NuPage; Life Technologies) in MOPS running buffer and subjected to immunoblotting.

Flow Cytometry Analysis

Human blood primary immune cells were obtained from healthy donors. Ethical approval was obtained from the Oxfordshire Research Ethics Committee (Reference 09/H0606/5), and informed written consent was given by all donors. L18-MDP stimulation assay was performed as previously described (Ammann et al., 2014). In brief, healthy donor peripheral blood mononuclear cells (PBMC) were isolated by gradient centrifugation and cultured in RPMI1640 supplemented with 10% fetal calf serum. To enrich for monocytes, PBMC (2.5×10^6 cells) were plated in six-well plates and rested overnight. The following day, cells were gently washed with PBS and pre-incubated for 60 min with indicated concentrations of inhibitors followed by receptor activation with 200 ng/ml L18-MDP (InvivoGen) or 200 ng/ml LPS (Enzo Life Sciences) in the presence of Golgiplug (BD Biosciences). After 2.5 hr of stimulation, cells were harvested by scraping, put on ice, and stained with fixable viability dye (eBioscience) and surface monocyte markers. Following fixation and cell permeabilization (Cytofix/Cytoperm Kit; BD Biosciences) cells were stained for intracellular production of TNF. The following antibodies were used: anti-TNF-α (clone MAb11; eBioscience), anti-CD14 (clone M5E2; BioLegend), and anti-HLA-DR (clone L243; BioLegend). Results were acquired by flow cytometry (LSRFortessa; BD Biosciences) and data were analyzed using FlowJo software (version 10.0.6; Treestar).

SUPPLEMENTAL INFORMATION

Supplemental Information includes Supplemental Experimental Procedures, seven figures, and two tables and can be found with this article online at http://dx.doi.org/10.1016/j.chembiol.2015.07.017.

AUTHOR CONTRIBUTIONS

A.N.B., A.D., M.G.-H., and H.H.U. designed the research. P.C., Q.R., T.S., M.H., J.L.M., and D.S. performed the research and analyzed the data. S.R. and P.E.B. performed molecular docking studies. C.S. and G.D.C. conceived and synthesized the inhibitor CS6. P.C., T.S., H.H.U., M.G.-H., A.D., and A.N.B. wrote the paper.

ACKNOWLEDGMENTS

The authors would like to thank the Diamond Light Source for beamtime (proposal mx8421), as well as the staff of beamline I04 for assistance with crystal testing and data collection. H.H.U. is supported by the Crohn's & Colitis Foundation of America (CCFA) and by the Leona M. and Harry B. Helmsley Charitable Trust to study VEOIBD. T.S. is supported by the Deutsche Forschungsgemeinschaft (SCHW1730/1-1). A.D. is supported by National Institute of General Medical Sciences grants R01GM080356 and R01GM084205. M.G.-H. is supported by a Wellcome Trust Fellowship (102894/Z/13/Z), Ludwig Cancer Research, a Sapere Aude: Danish Council for independent Research-Starting Grant, and the EMBO Young Investigator Programme. The SGC is a registered charity (no. 1097737) that receives funds from AbbVie, Bayer, Boehringer Ingelheim, Genome Canada through Ontario Genomics Institute Grant OGI-055, GlaxoSmithKline, Janssen, Lilly Canada, the Novartis Research Foundation, the Ontario Ministry of Economic Development and Innovation, Pfizer, Takeda, and Wellcome Trust Grant 092809/Z/10/Z.

Received: March 18, 2015
Revised: July 1, 2015
Accepted: July 24, 2015
Published: August 27, 2015

REFERENCES

Ammann, S., Elling, R., Gyrd-Hansen, M., Duckers, G., Bredius, R., Burns, S.O., Edgar, J.D., Worth, A., Brandau, H., Warnatz, K., et al. (2014). A new functional assay for the diagnosis of X-linked inhibitor of apoptosis (XIAP) deficiency. Clin. Exp. Immunol. *176*, 394–400.

Andraos, R., Qian, Z., Bonenfant, D., Rubert, J., Vangrevelinghe, E., Scheufler, C., Marque, F., Regnier, C.H., De Pover, A., Ryckelynck, H., et al. (2012). Modulation of activation-loop phosphorylation by JAK inhibitors is binding mode dependent. Cancer Discov. *2*, 512–523.

Argast, G.M., Fausto, N., and Campbell, J.S. (2005). Inhibition of RIP2/RIck/CARDIAK activity by pyridinyl imidazole inhibitors of p38 MAPK. Mol. Cell. Biochem. *268*, 129–140.

Bertrand, M.J., Lippens, S., Staes, A., Gilbert, B., Roelandt, R., De Medts, J., Gevaert, K., Declercq, W., and Vandenabeele, P. (2011). cIAP1/2 are direct E3 ligases conjugating diverse types of ubiquitin chains to receptor interacting proteins kinases 1 to 4 (RIP1-4). PLoS One *6*, e22356.

Caruso, R., Warner, N., Inohara, N., and Nunez, G. (2014). NOD1 and NOD2: signaling, host defense, and inflammatory disease. Immunity *41*, 898–908.

Chamaillard, M., Hashimoto, M., Horie, Y., Masumoto, J., Qiu, S., Saab, L., Ogura, Y., Kawasaki, A., Fukase, K., Kusumoto, S., et al. (2003). An essential role for NOD1 in host recognition of bacterial peptidoglycan containing diaminopimelic acid. Nat. Immunol. *4*, 702–707.

Cooney, R., Baker, J., Brain, O., Danis, B., Pichulik, T., Allan, P., Ferguson, D.J., Campbell, B.J., Jewell, D., and Simmons, A. (2010). NOD2 stimulation induces autophagy in dendritic cells influencing bacterial handling and antigen presentation. Nat. Med. *16*, 90–97.

Cortes, J.E., Kim, D.W., Pinilla-Ibarz, J., le Coutre, P., Paquette, R., Chuah, C., Nicolini, F.E., Apperley, J.F., Khoury, H.J., Talpaz, M., et al. (2013). A phase 2 trial of ponatinib in Philadelphia chromosome-positive leukemias. N. Engl. J. Med. *369*, 1783–1796.

Couturier-Maillard, A., Secher, T., Rehman, A., Normand, S., De Arcangelis, A., Haesler, R., Huot, L., Grandjean, T., Bressenot, A., Delanoye-Crespin, A., et al. (2013). NOD2-mediated dysbiosis predisposes mice to transmissible colitis and colorectal cancer. J. Clin. Invest. *123*, 700–711.

Cramer, P., and Muller, C.W. (1999). A firm hand on NFkappaB: structures of the IkappaBalpha-NFkappaB complex. Structure *7*, R1–R6.

Damgaard, R.B., Nachbur, U., Yabal, M., Wong, W.W., Fiil, B.K., Kastirr, M., Rieser, E., Rickard, J.A., Bankovacki, A., Peschel, C., et al. (2012). The ubiquitin ligase XIAP recruits LUBAC for NOD2 signaling in inflammation and innate immunity. Mol. Cell *46*, 746–758.

Damgaard, R.B., Fiil, B.K., Speckmann, C., Yabal, M., zur Stadt, U., Bekker-Jensen, S., Jost, P.J., Ehl, S., Mailand, N., and Gyrd-Hansen, M. (2013). Disease-causing mutations in the XIAP BIR2 domain impair NOD2-dependent immune signalling. EMBO Mol. Med. *5*, 1278–1295.

Dorsch, M., Wang, A., Cheng, H., Lu, C., Bielecki, A., Charron, K., Clauser, K., Ren, H., Polakiewicz, R.D., Parsons, T., et al. (2006). Identification of a regulatory autophosphorylation site in the serine-threonine kinase RIP2. Cell. Signal. *18*, 2223–2229.

Elinav, E., Nowarski, R., Thaiss, C.A., Hu, B., Jin, C., and Flavell, R.A. (2013). Inflammation-induced cancer: crosstalk between tumours, immune cells and microorganisms. Nat. Rev. Cancer *13*, 759–771.

Fiil, B.K., Damgaard, R.B., Wagner, S.A., Keusekotten, K., Fritsch, M., Bekker-Jensen, S., Mailand, N., Choudhary, C., Komander, D., and Gyrd-Hansen, M. (2013). OTULIN restricts Met1-linked ubiquitination to control innate immune signaling. Mol. Cell *50*, 818–830.

Girardin, S.E., Boneca, I.G., Carneiro, L.A., Antignac, A., Jehanno, M., Viala, J., Tedin, K., Taha, M.K., Labigne, A., Zahringer, U., et al. (2003a). Nod1 detects a unique muropeptide from gram-negative bacterial peptidoglycan. Science *300*, 1584–1587.

Girardin, S.E., Boneca, I.G., Viala, J., Chamaillard, M., Labigne, A., Thomas, G., Philpott, D.J., and Sansonetti, P.J. (2003b). Nod2 is a general sensor of peptidoglycan through muramyl dipeptide (MDP) detection. J. Biol. Chem. *278*, 8869–8872.

Goh, F.Y., Cook, K.L., Upton, N., Tao, L., Lah, L.C., Leung, B.P., and Wong, W.S. (2013). Receptor-interacting protein 2 gene silencing attenuates allergic airway inflammation. J. Immunol. *191*, 2691–2699.

Hollenbach, E., Vieth, M., Roessner, A., Neumann, M., Malfertheiner, P., and Naumann, M. (2005). Inhibition of RICK/nuclear factor-kappaB and p38 signaling attenuates the inflammatory response in a murine model of Crohn disease. J. Biol. Chem. *280*, 14981–14988.

Homer, C.R., Kabi, A., Marina-Garcia, N., Sreekumar, A., Nesvizhskii, A.I., Nickerson, K.P., Chinnaiyan, A.M., Nunez, G., and McDonald, C. (2012). A dual role for receptor-interacting protein kinase 2 (RIP2) kinase activity in nucleotide-binding oligomerization domain 2 (NOD2)-dependent autophagy. J. Biol. Chem. *287*, 25565–25576.

Huang, W.S., Metcalf, C.A., Sundaramoorthi, R., Wang, Y., Zou, D., Thomas, R.M., Zhu, X., Cai, L., Wen, D., Liu, S., et al. (2010). Discovery of 3-[2-(imidazo[1,2-b]pyridazin-3-yl)ethynyl]-4-methyl-N-{4-[(4-methylpiperazin-1-y l)methyl]-3-(trifluoromethyl)phenyl}benzamide (AP24534), a potent, orally active pan-inhibitor of breakpoint cluster region-abelson (BCR-ABL) kinase including the T315I gatekeeper mutant. J. Med. Chem. *53*, 4701–4719.

Hugot, J.P., Chamaillard, M., Zouali, H., Lesage, S., Cezard, J.P., Belaiche, J., Almer, S., Tysk, C., O'Morain, C.A., Gassull, M., et al. (2001). Association of NOD2 leucine-rich repeat variants with susceptibility to Crohn's disease. Nature *411*, 599–603.

Inohara, N., Koseki, T., del Peso, L., Hu, Y., Yee, C., Chen, S., Carrio, R., Merino, J., Liu, D., Ni, J., et al. (1999). Nod1, an Apaf-1-like activator of caspase-9 and nuclear factor-kappaB. J. Biol. Chem. *274*, 14560–14567.

Inohara, N., Ogura, Y., Fontalba, A., Gutierrez, O., Pons, F., Crespo, J., Fukase, K., Inamura, S., Kusumoto, S., Hashimoto, M., et al. (2003). Host recognition of bacterial muramyl dipeptide mediated through NOD2. Implications for Crohn's disease. J. Biol. Chem. *278*, 5509–5512.

Jostins, L., Ripke, S., Weersma, R.K., Duerr, R.H., McGovern, D.P., Hui, K.Y., Lee, J.C., Schumm, L.P., Sharma, Y., Anderson, C.A., et al. (2012). Host-microbe interactions have shaped the genetic architecture of inflammatory bowel disease. Nature *491*, 119–124.

Jun, J.C., Cominelli, F., and Abbott, D.W. (2013). RIP2 activity in inflammatory disease and implications for novel therapeutics. J. Leukoc. Biol. *94*, 927–932.

Kim, J.Y., Omori, E., Matsumoto, K., Nunez, G., and Ninomiya-Tsuji, J. (2008). TAK1 is a central mediator of NOD2 signaling in epidermal cells. J. Biol. Chem. *283*, 137–144.

Lee, T.H., Shank, J., Cusson, N., and Kelliher, M.A. (2004). The kinase activity of Rip1 is not required for tumor necrosis factor-alpha-induced IkappaB kinase or p38 MAP kinase activation or for the ubiquitination of Rip1 by Traf2. J. Biol. Chem. *279*, 33185–33191.

Mayle, S., Boyle, J.P., Sekine, E., Zurek, B., Kufer, T.A., and Monie, T.P. (2014). Engagement of nucleotide-binding oligomerization domain-containing protein 1 (NOD1) by receptor-interacting protein 2 (RIP2) is insufficient for signal transduction. J. Biol. Chem. *289*, 22900–22914.

Moore, M., Hirte, H.W., Siu, L., Oza, A., Hotte, S.J., Petrenciuc, O., Cihon, F., Lathia, C., and Schwartz, B. (2005). Phase I study to determine the safety and pharmacokinetics of the novel Raf kinase and VEGFR inhibitor BAY 43-9006, administered for 28 days on/7 days off in patients with advanced, refractory solid tumors. Ann. Oncol. *16*, 1688–1694.

Mross, K., Frost, A., Steinbild, S., Hedbom, S., Buchert, M., Fasol, U., Unger, C., Kratzschmar, J., Heinig, R., Boix, O., et al. (2012). A phase I dose-escalation study of regorafenib (BAY 73-4506), an inhibitor of oncogenic, angiogenic, and stromal kinases, in patients with advanced solid tumors. Clin. Cancer Res. *18*, 2658–2667.

Nachbur, U., Stafford, C.A., Bankovacki, A., Zhan, Y., Lindqvist, L.M., Fiil, B.K., Khakham, Y., Ko, H.J., Sandow, J.J., Falk, H., et al. (2015). A RIPK2 inhibitor delays NOD signalling events yet prevents inflammatory cytokine production. Nat. Commun. *6*, 6442.

Najjar, M., Suebsuwong, C., Ray, S.S., Thapa, R.J., Maki, J.L., Nogusa, S., Shah, S., Saleh, D., Gough, P.J., Bertin, J., et al. (2015). Structure guided design of potent and selective ponatinib-based hybrid inhibitors for RIPK1. Cell Rep. *10*, 1850–1860.

Negroni, A., Stronati, L., Pierdomenico, M., Tirindelli, D., Di Nardo, G., Mancini, V., Maiella, G., and Cucchiara, S. (2009). Activation of NOD2-mediated intestinal pathway in a pediatric population with Crohn's disease. Inflamm. Bowel Dis. *15*, 1145–1154.

Nembrini, C., Kisielow, J., Shamshiev, A.T., Tortola, L., Coyle, A.J., Kopf, M., and Marsland, B.J. (2009). The kinase activity of Rip2 determines its stability and consequently Nod1- and Nod2-mediated immune responses. J. Biol. Chem. *284*, 19183–19188.

Niesen, F.H., Berglund, H., and Vedadi, M. (2007). The use of differential scanning fluorimetry to detect ligand interactions that promote protein stability. Nat. Protoc. *2*, 2212–2221.

Ogura, Y., Bonen, D.K., Inohara, N., Nicolae, D.L., Chen, F.F., Ramos, R., Britton, H., Moran, T., Karaliuskas, R., Duerr, R.H., et al. (2001a). A frameshift mutation in NOD2 associated with susceptibility to Crohn's disease. Nature *411*, 603–606.

Ogura, Y., Inohara, N., Benito, A., Chen, F.F., Yamaoka, S., and Nunez, G. (2001b). Nod2, a Nod1/Apaf-1 family member that is restricted to monocytes and activates NF-kappaB. J. Biol. Chem. *276*, 4812–4818.

Park, J.H., Kim, Y.G., McDonald, C., Kanneganti, T.D., Hasegawa, M., Body-Malapel, M., Inohara, N., and Nunez, G. (2007). RICK/RIP2 mediates innate immune responses induced through Nod1 and Nod2 but not TLRs. J. Immunol. *178*, 2380–2386.

Philpott, D.J., Sorbara, M.T., Robertson, S.J., Croitoru, K., and Girardin, S.E. (2014). NOD proteins: regulators of inflammation in health and disease. Nat. Rev. Immunol. *14*, 9–23.

Saxena, M., and Yeretssian, G. (2014). NOD-like receptors: master regulators of inflammation and cancer. Front. Immunol. *5*, 327.

Sebastian, S., Hernandez, V., Myrelid, P., Kariv, R., Tsianos, E., Toruner, M., Marti-Gallostra, M., Spinelli, A., van der Meulen-de Jong, A.E., Yuksel, E.S., et al. (2014). Colorectal cancer in inflammatory bowel disease: results of the 3rd ECCO pathogenesis scientific workshop (I). J. Crohns Colitis *8*, 5–18.

Shaw, P.J., Barr, M.J., Lukens, J.R., McGargill, M.A., Chi, H., Mak, T.W., and Kanneganti, T.D. (2011). Signaling via the RIP2 adaptor protein in central nervous system-infiltrating dendritic cells promotes inflammation and autoimmunity. Immunity *34*, 75–84.

Sorbara, M.T., Ellison, L.K., Ramjeet, M., Travassos, L.H., Jones, N.L., Girardin, S.E., and Philpott, D.J. (2013). The protein ATG16L1 suppresses inflammatory cytokines induced by the intracellular sensors Nod1 and Nod2 in an autophagy-independent manner. Immunity *39*, 858–873.

Tao, M., Scacheri, P.C., Marinis, J.M., Harhaj, E.W., Matesic, L.E., and Abbott, D.W. (2009). ITCH K63-ubiquitinates the NOD2 binding protein, RIP2, to influence inflammatory signaling pathways. Curr. Biol. *19*, 1255–1263.

Tigno-Aranjuez, J.T., Asara, J.M., and Abbott, D.W. (2010). Inhibition of RIP2's tyrosine kinase activity limits NOD2-driven cytokine responses. Genes Dev. *24*, 2666–2677.

Tigno-Aranjuez, J.T., Bai, X., and Abbott, D.W. (2013). A discrete ubiquitin-mediated network regulates the strength of NOD2 signaling. Mol. Cell. Biol. *33*, 146–158.

Tigno-Aranjuez, J.T., Benderitter, P., Rombouts, F., Deroose, F., Bai, X., Mattioli, B., Cominelli, F., Pizarro, T.T., Hoflack, J., and Abbott, D.W. (2014). In vivo inhibition of RIPK2 kinase alleviates inflammatory disease. J. Biol. Chem. *289*, 29651–29664.

Vogt, J., Traynor, R., and Sapkota, G.P. (2011). The specificities of small molecule inhibitors of the TGFβ and BMP pathways. Cell. Signal. *23*, 1831–1842.

Xie, T., Peng, W., Liu, Y., Yan, C., Maki, J., Degterev, A., Yuan, J., and Shi, Y. (2013a). Structural basis of RIP1 inhibition by necrostatins. Structure *21*, 493–499.

Xie, T., Peng, W., Yan, C., Wu, J., Gong, X., and Shi, Y. (2013b). Structural insights into RIP3-mediated necroptotic signaling. Cell Rep. *5*, 70–78.

Yang, Y., Yin, C., Pandey, A., Abbott, D., Sassetti, C., and Kelliher, M.A. (2007). NOD2 pathway activation by MDP or *Mycobacterium tuberculosis* infection involves the stable polyubiquitination of Rip2. J. Biol. Chem. *282*, 36223–36229.

Yang, S., Wang, B., Humphries, F., Jackson, R., Healy, M.E., Bergin, R., Aviello, G., Hall, B., McNamara, D., Darby, T., et al. (2013). Pellino3 ubiquitinates RIP2 and mediates Nod2-induced signaling and protective effects in colitis. Nat. Immunol. *14*, 927–936.

Zhao, Z., Wu, H., Wang, L., Liu, Y., Knapp, S., Liu, Q., and Gray, N.S. (2014). Exploration of type II binding mode: a privileged approach for kinase inhibitor focused drug discovery? ACS Chem. Biol. *9*, 1230–1241.

Chemistry & Biology, Volume *22*

Supplemental Information

Inflammatory Signaling by NOD-RIPK2 Is Inhibited by Clinically Relevant Type II Kinase Inhibitors

Peter Canning, Qui Ruan, Tobias Schwerd, Matous Hrdinka, Jenny L. Maki, Danish Saleh, Chalada Suebsuwong, Soumya Ray, Paul E. Brennan, Gregory D. Cuny, Holm H. Uhlig, Mads Gyrd-Hansen, Alexei Degterev, and Alex N. Bullock

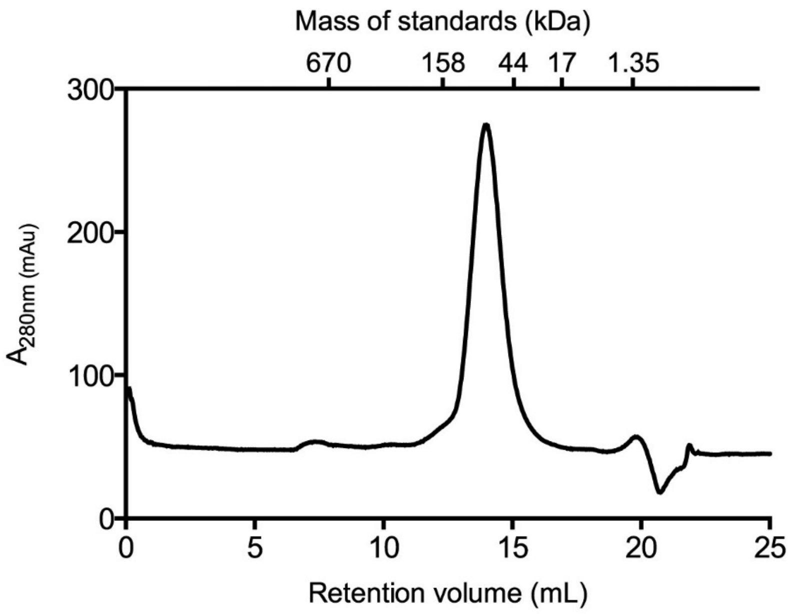

Figure S1, Related to Figure 2. Dimerization of RIPK2 in solution. Analytical gel filtration trace of RIPK2. Some 500 µL of purified RIPK2 at 50 µM concentration was injected onto a superdex 200 10/300 GL column and monitored by absorbance at 280 nm (A280). The retention volume of RIPK2 was compared with the retention volumes of gel filtration MW standards and indicated that the protein is a dimer in solution.

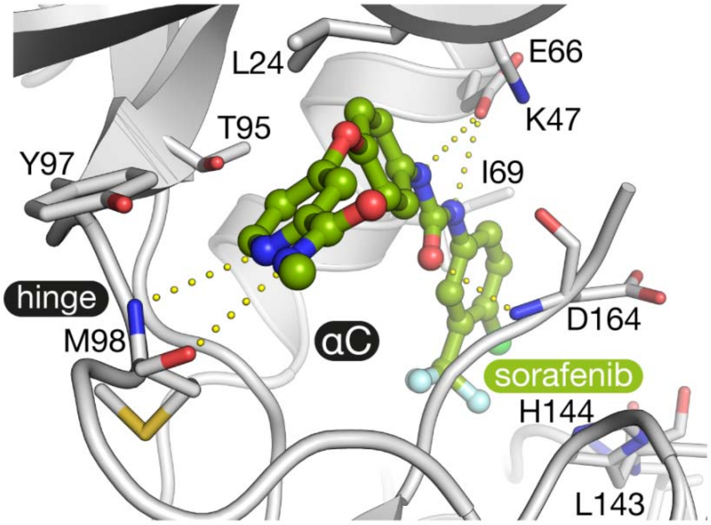

Figure S2, Related to Figure 3. Predicted binding mode of sorafenib. The main chain is shown as a cartoon representation and the side-chains of selected residues shown as sticks with residue numbers. Hydrogen bonds formed between RIPK2 and bound inhibitor are shown as dashed lines. Docking was performed with ICM-Pro (Molsoft).

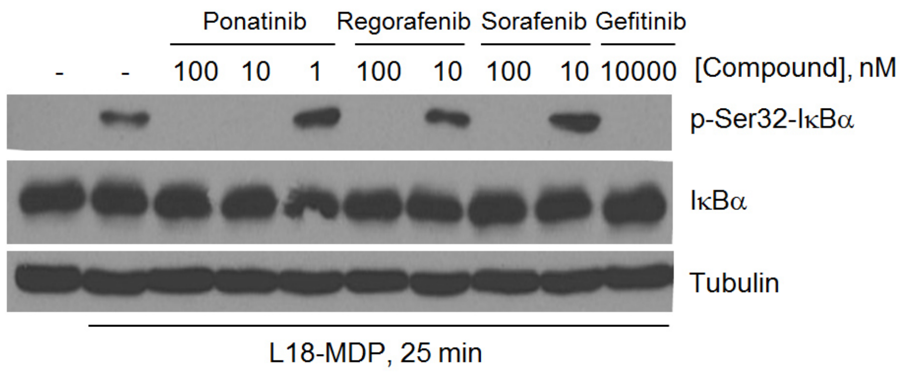

Figure S3, Related to Figure 4. Inhibitors of RIPK2 block IκBα phosphorylation in a dose-dependent manner. Phosphorylation of IκBα in HEKBlue cells. Cells were treated with indicated concentrations of inhibitors, followed 30 minutes later by stimulation with 1 μg/mL L18-MDP. Cells were harvested after 25 minutes and changes in IκBα protein phosphorylation were analyzed by Western blotting. Levels of tubulin were used as a loading control.

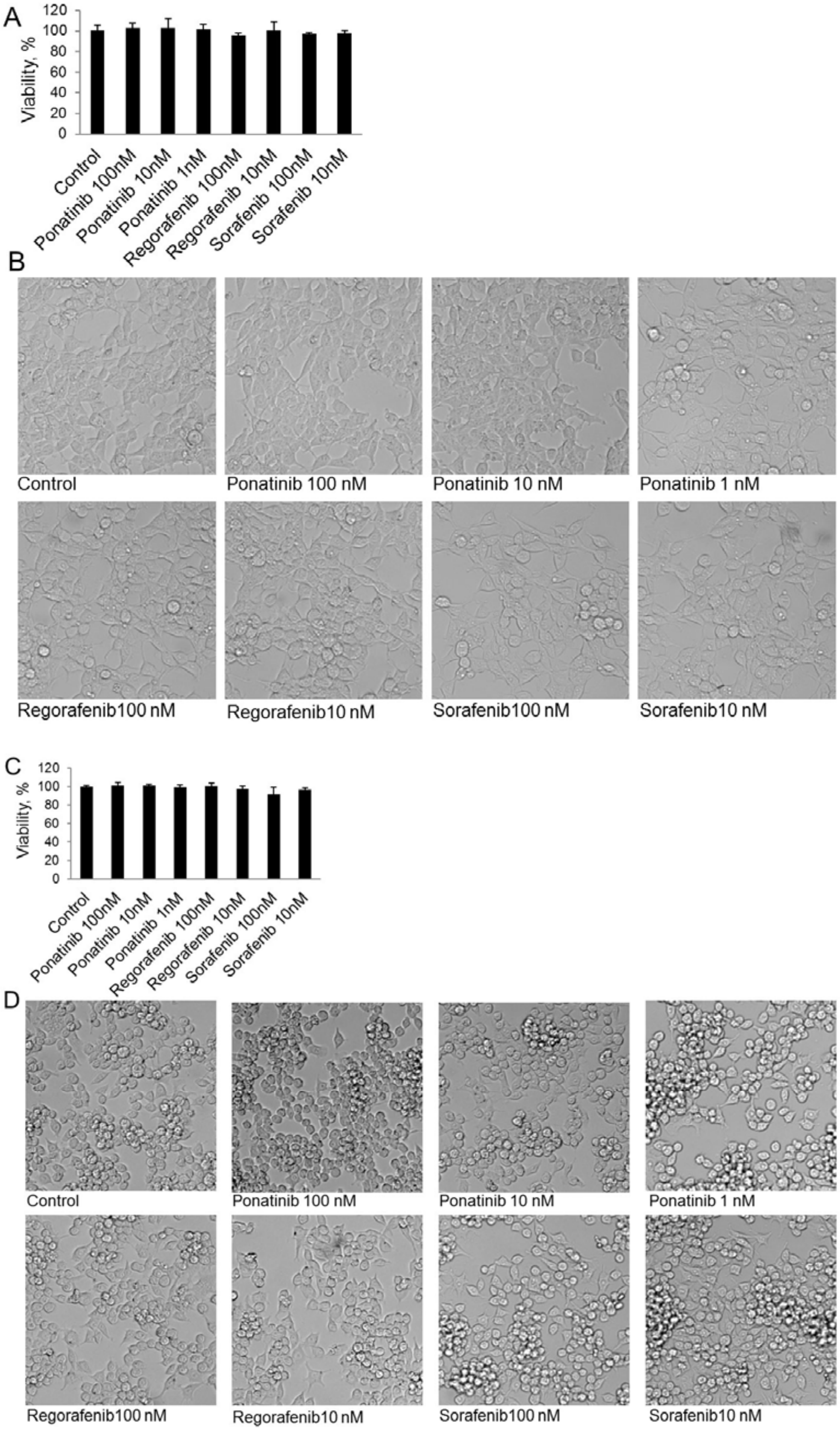
A
Viability, %
0
20
40
60
80
100
120
Control
Ponatinib 100nM
Ponatinib 10nM
Ponatinib 1nM
Regorafenib 100nM
Regorafenib 10nM
Sorafenib 100nM
Sorafenib 10nM
B
Control
Ponatinib 100 nM
Ponatinib 10 nM
Ponatinib 1 nM
Regorafenib100 nM
Regorafenib10 nM
Sorafenib100 nM
Sorafenib10 nM
C
Viability, %
0
20
40
60
80
100
120
Control
Ponatinib 100nM
Ponatinib 10nM
Ponatinib 1nM
Regorafenib 100nM
Regorafenib 10nM
Sorafenib 100nM
Sorafenib 10nM
D
Control
Ponatinib 100 nM
Ponatinib 10 nM
Ponatinib 1 nM
Regorafenib100 nM
Regorafenib10 nM
Sorafenib100 nM
Sorafenib10 nM

Figure S4, Related to Figure 4 and Figure 6. Inhibitors of RIPK2 do not impact cell viability or morphology. Cells were treated with the indicated concentrations of the inhibitors for 24 hours. (B) Cell viability in HEKBlue cells. (C) Morphology of HEKBlue cells. (D) Cell viability of RAW264.7cells. (E) Morphology of RAW264.7 cells. None of the inhibitors affected the viability or morphological appearance of RAW264.7 cells, except for 100 nM ponatinib, which caused rounding of RAW cells and likely reflects off target activity at the highest concentration. Experiments were performed in triplicate, error bars indicate SD values.

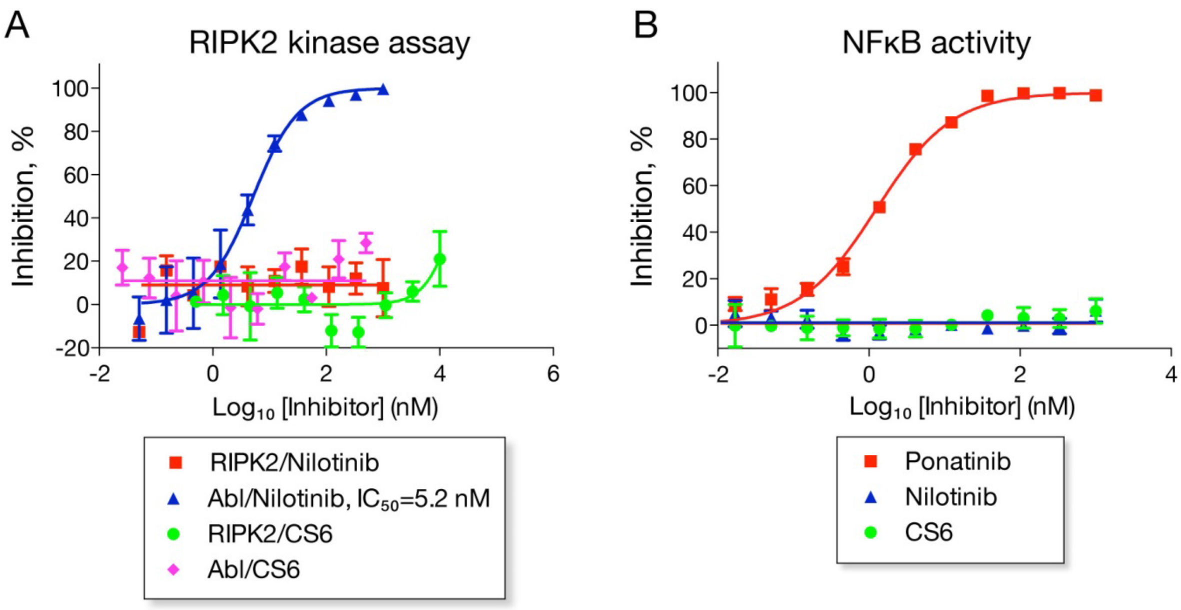

Figure S5, Related to Figure 3 and Figure 4. Analysis of Abl and RIPK2 inhibition by nilotinib and CS6. (A) Dose-response curves for RIPK2 inhibition by nilotinib and CS6. Experiments were performed in duplicate. *In vitro* kinase activity was measured using the ADPGlo assay. Non-linear curve fitting to calculate IC50 values was performed using Prism software. Experiments were performed in duplicate, error bars indicate SD values. (B) Inhibition of NFκB activation in HEKBlue cells. HEKBlue reporter cells, expressing NOD2 and NFκB-SEAP reporter were treated with 6-8 concentrations of each inhibitor in triplicate followed by stimulation with 1 μg/mL L18-MDP for 8 hours. SEAP activity was detected using HEKBlue media with detection of absorbance at 620 nM in a Wallac3V plate reader. Non-linear curve fitting to calculate EC50 values was performed using Prism software. Experiments were performed in triplicate, error bars indicate SD values.

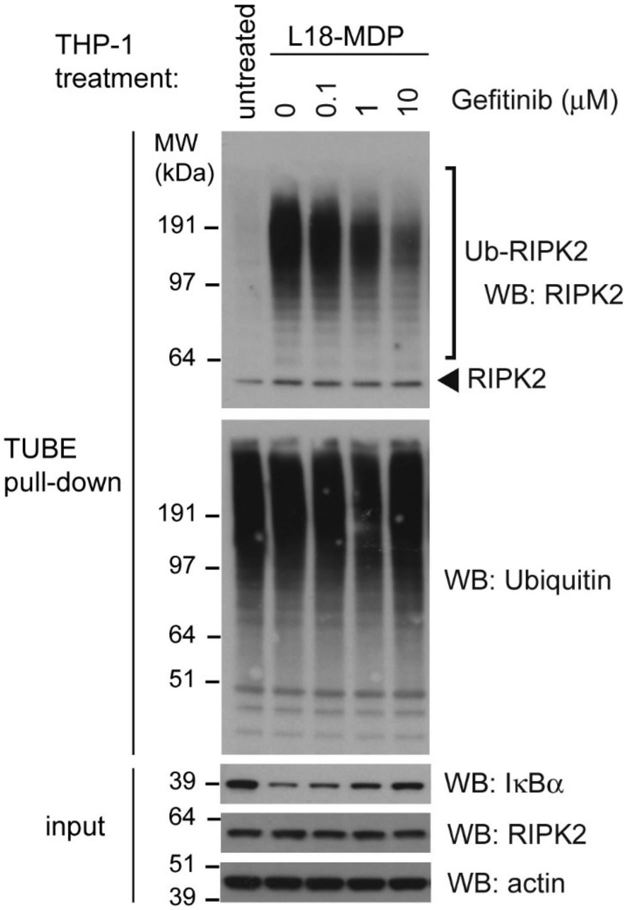

Figure S6, Related to Figure 5. Inhibition of NOD2-dependent ubiquitination. RIPK2 ubiquitination in THP-1 cells upon L18-MDP stimulation (1 hour) in the presence of increasing concentrations of gefitinib. Representative result of the experiment performed three times.

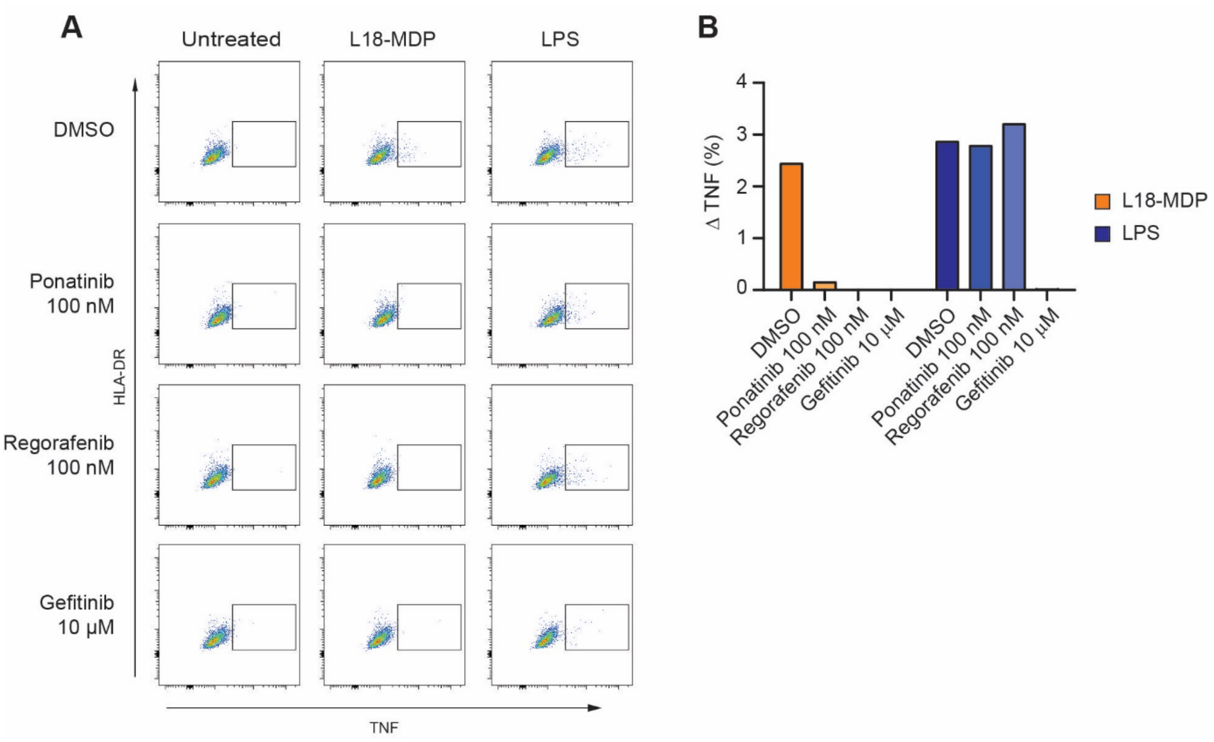

Figure S7, Related to Figure 7. Inhibition of NOD2 signaling in the human monocytic cell line THP-1. Representative FACS blots (A) and quantification of TNF production (B) in THP-1 cells stimulated with L18-MDP or LPS. In indicated conditions cells were preincubated with kinase inhibitors for 1 hour before activation of NOD2 and TLR4 receptor for 2.5 hours.

Supplemental Data

Table S1, Related to Figure 1 Thermal Shift Inhibitor Screening for RIPK2

Rank	Tm shift (°C)	Inhibitor	Supplier	Supplier I.D.
1	23.1	Ponatinib (AP24534)	Selleck Chemicals	S1490
2	12.1	LDN-193189	Paul Yu, Harvard	N/A
3	10.0	K252a	Calbiochem (EMD)	420298
4	9.6	Dorsomorphin	Sigma	P5499
5	9.5	Dasatinib	Sequoia	863127-77-9
6	9.5	Gefitinib	Biaffin GmbH	PKI-GFTB-010
7	9.1	Staurosporine	AXXORA	S-9300
8	8.9	Raf1 kinase inhibitor II	Calbiochem (EMD)	553011
9	8.7	BIBX1382	Calbiochem (EMD)	324832
10	8.4	TGFβ inhibitor IV (A-83-01)	Calbiochem (EMD)	616454

Table S2, Related to Figure 1 Diffraction data collection and refinement statistics

**Data collection**	
Wavelength (Å)	0.9795
Resolution range (Å)	58.74 - 2.75 (2.9 - 2.75)
Space group	$P2_12_12_1$
Cell dimensions	
a, b, c (Å)	58.7, 86.7, 137.3
α, β, γ (°)	90, 90, 90
Total reflections	136,896 (19978)
Unique reflections	18905 (2690)
Multiplicity	7.2 (7.4)
Completeness (%)	100 (100.00)
Mean $(I)/\sigma(I)$	12.1 (2)
Wilson *B*-factor ($Å^2$)	63.32
$R_{p.i.m}$	0.066 (0.557)
$CC_{1/2}$	0.998 (0.759)
**Refinement**	
R-work	0.2006 (0.297)
R-free	0.2436 (0.295)
Number of atoms	4445
macromolecules	4318
ligand	78
water	45
Protein residues	560
R.M.S. deviations	
Bond lengths (Å)	0.01
Bond angles (°)	1.295
Ramachandran favoured (%)	96
Ramachandran outliers (%)	0
MolProbity clashscore	4.58
Avg *B*-factors ($Å^2$)	63.7
macromolecules	64.2
solvent	48.6
ligand	46

Values in brackets show the statistics for the highest resolution shells.
R.M.S indicates root-mean-square.

Supplemental Experimental Procedures

Antibodies and Reagents

Commercially available antibodies for immunoblotting were obtained from the following sources: mouse anti-RIPK1 (clone 38/RIP) from BD BioSciences, mouse anti-RIPK2 (clone A-10) and rabbit RIPK2 (clone H-300) from Santa Cruz, mouse anti-Ubiquitin (clone Ubi-1, Imgenex), rabbit anti-phospho-Ser176-RIPK2 (Cell Signaling), mouse anti-α-tubulin (clone DM1A, Cell Signaling), rabbit anti-phospho-Thr202/Tyr204 Erk1/2 (clone D13.14.4E, Cell Signaling), mouse anti-phospho-Tyr (clone P-Tyr-100, Cell Signaling), mouse anti-IκBα (clone L35A5, Cell Signaling), and anti-actin (clone MAB1501, Millipore). The NOD1 ligand Tri-DAP, NOD2 ligands L18-MDP and MDP and TLR2 ligand Pam3CSK4 were purchased from Invivogen, LPS (*E.coli* 0111:B4) from Sigma and recombinant TNF from Enzo.

Cells

THP-1 cells were cultured in RPMI 1640 medium (Gibco) supplemented with 10% FBS and Penicillin / Streptomycin at density 0.6 −1.0 million cells per mL. RAW264.7 cells were maintained in DMEM medium (Fisher) supplemented with 10% FBS (Sigma) and 1% antibiotic-antimycotic mix (PSA) (Invitrogen). HEK-Blue hNOD2 cells (Invivogen) were maintained in DMEM/10% FBS/PSA supplemented with Normocin (100 µg/mL), Blasticidin (30 µg/mL) and Zeocin (100 µg/mL).

Western blot analysis

HEK-Blue cells were seeded into 10 cm^2 dishes to achieve 80-90% confluency after 48 hr. Cells were pre-treated with inhibitors for 30 min and stimulated with L18-MDP for 30 min. Cells were lysed in RIPA buffer supplemented with PMSF (Cell Signaling), briefly sonicated and spun in

4^0C centrifuge at 14,000 rpm for 15 min to collect lysates. Protein concentrations were measured using 660 nM protein assay reagent (Pierce). Equal amounts of proteins were separated using 8-10% SDS-PAGE, followed by overnight incubations with antibodies according to the manufacturer's recommendations.

ADPGlo *in vitro* kinase assays

For ADPGlo (Promega) assays, 1 ng Abl or 10 ng of RIPK2 was diluted in reaction buffer (40 mM Tris-HCl pH 7.5, 20 mM $MgCl_2$, 0.5 mM DTT, 0.01% BSA) supplemented with 50 μM ATP and 10-point dose range of inhibitors. Reactions were performed at room temperature for 1 hour. Reactions were performed in 5 μL total volume (5% final concentration of DMSO) and stopped by addition of 5 μL of ADPGlo reagent for 40 min at room temperature. Luminescent signal was generated by addition of 10 μL of kinase detection reagent for 30 minutes at room temperature and determined using Victor3V platereader (Perkin Elmer). Specific signal was calculated by subtracting values in the wells without protein and inhibitor from the values in the test wells. Inhibition, % = ((specific signal (DMSO control) - specific signal (inhibitor))/ (specific signal (DMSO control))) x 100%. Non-linear regression to determine IC_{50} values was performed using Prism software (GraphPad).

qPCR analysis in RAW264.7 cells

RAW cells were seeded at $2X10^5$ cells/well in 12 well plates. After 24 hours, cells were treated with inhibitors and stimulated with 10 μg/mL MDP for 24 hours. Total RNAs were isolated using ZR RNA miniprep kit. 1 μg of total RNA was used to synthesize cDNA using iScript cDNA synthesis kit (Bio-Rad). cDNAs were diluted 5-fold with water and 2 μL were used in 20 μL qPCR reactions including 10 μL 2X VeriQuest SYBR mix (Affymetrix) and 1 μL of 10 μM primers. Cycling parameters were: 50°C – 2 min, 95°C – 10 min, 45 cycles: 95°C – 15 sec,

60°C – 30 sec. Reactions were performed using a Roche480 machine. GAPDH was used as a house keeping control to normalize mRNA values. The following primers were used: *mGAPDH* forward 5'-TGTGTCCGTCGTGGATCTGA-3'; *mGAPDH* reverse 5'-GGTCCTCAGTGTAGCCCAAG-3'; *mCCL4* forward 5'-TTCCTGCTGTTTCTCTTACACCT-3'; *mCCL3* reverse 5'-CTGTCTGCCTCTTTTGGTCAG-3'; *mCXCL2* forward 5'-CCAACCACCAGGCTAGAGG-3'; *mCXCL2* reverse 5'-GCGTCACACTCAAGCTCTG-3'; *mRANTES* forward 5'-TTTGCCTACCTCTCCCTCG-3'; *mRANTES* reverse 5'-CGACTGCAAGATTGGAGCACT-3'.

Purification of RIPK2

DNA encoding the kinase domain of human *RIPK2* (Uniprot: O43353, residues 8-317) was cloned into the transfer vector pFB-LIC-Bse, which encodes for an N-terminal hexahistidine tag and a Tobacco Etch Virus Protease A (TEV) cleavage site. Bacmid DNA was prepared in *E. coli* strain DH10Bac and used to generate baculoviruses in Sf9 insect cells. For expression, viruses were used to Sf9 infect cells grown in Insect-Xpress media (Lonza) to a density of $2x10^6$ cells/mL. Cells were harvested after 48 hours by centrifugation and resuspended in 15 mL binding buffer (50 mM HEPES pH 7.4, 500 mM NaCl, 5% glycerol, 5 mM Imidazole) supplemented with protease inhibitor cocktail set V (Calbiochem) at 1:1000 dilution. Cells were lysed using an Emulsiflex C5 homogeniser, clarified by centrifugation, and the recombinant protein collected by nickel-affinity chromatography and eluted by imidazole. RIPK2 protein was treated with lambda phosphatase overnight at 4°C before further purification on a Superdex 75 26/60 gel filtration column pre-equilibrated in 10 mM HEPES pH 7.4, 500 mM NaCl, 5% glycerol, 1 mM tris(2-carboxyethyl)phosphine (TCEP). The final protein was supplemented with 5 mM L-arginine, 5 mM L-glutamate and 2 mM DTT. DNA sequencing and mass spectrometry identified an Arg171Cys mutation in the kinase activation loop.

Crystallization and structure determination

RIPK2 was concentrated to 3.7 mg/mL and ponatinib added to a slight molar excess. Crystals were grown using the vapour-diffusion technique in 150 nL sitting drops containing 50 nL protein and 100 nL of a reservoir solution containing 0.1 M ammonium citrate and 16% (w/v) PEG3350 at 20°C. Crystals were cryo-protected by addition of 25% ethylene glycol before being vitrified in liquid nitrogen. Diffraction data were collected at 100K on Diamond Light Source beamline I04. Data were indexed and integrated using XDS (Kabsch, 2010; Leslie and Powell, 2007) and scaled using AIMLESS (Evans and Murshudov, 2013) in the CCP4 suite of programs (Winn, et al., 2011). Phases were identified using molecular replacement in PHASER (McCoy, et al., 2007) and the PDB: 3PPZ as a search model. Structures were built using PHENIX.AUTOBUILD (Adams, et al., 2010) and then refined and modified using alternate rounds of REFMAC5 (Murshudov, et al., 2011) and COOT (Emsley, et al., 2010). TLS groups were determined using the TLSMD server (Painter and Merritt, 2006). The refined structure was validated with MolProbity (Chen, et al., 2010) and the atomic coordinate files deposited in the Protein Data Bank with Autodep (Yang, et al., 2004). Structure figures were prepared with PyMOL (Schrödinger LLC., Version 1.2r3pre), sequence alignments with ESPript (Robert and Gouet, 2014) and structure comparisons using PDBeFOLD (Krissinel and Henrick, 2004).

Supplemental References

Adams, P.D., Afonine, P.V., Bunkoczi, G., Chen, V.B., Davis, I.W., Echols, N., Headd, J.J., Hung, L.W., Kapral, G.J., Grosse-Kunstleve, R.W., et al. (2010). PHENIX: a comprehensive Python-based system for macromolecular structure solution. Acta Crystallogr D Biol Crystallogr 66, 213-221.

Chen, V.B., Arendall, W.B., 3rd, Headd, J.J., Keedy, D.A., Immormino, R.M., Kapral, G.J., Murray, L.W., Richardson, J.S., and Richardson, D.C. (2010). MolProbity: all-atom

structure validation for macromolecular crystallography. Acta Crystallogr D Biol Crystallogr 66, 12-21.

Emsley, P., Lohkamp, B., Scott, W.G., and Cowtan, K. (2010). Features and development of Coot. Acta Crystallogr D Biol Crystallogr 66, 486-501.

Evans, P.R., and Murshudov, G.N. (2013). How good are my data and what is the resolution? Acta Crystallogr D Biol Crystallogr 69, 1204-1214.

Kabsch, W. (2010). Xds. Acta Crystallogr D Biol Crystallogr 66, 125-132.

Krissinel, E., and Henrick, K. (2004). Secondary-structure matching (SSM), a new tool for fast protein structure alignment in three dimensions. Acta Crystallogr D Biol Crystallogr 60, 2256-2268.

Leslie, A.G.W., and Powell, H.R. (2007). Processing diffraction data with mosflm

Evolving Methods for Macromolecular Crystallography, R.J. Read, and J.L. Sussman, eds. (Springer Netherlands), pp. 41-51.

McCoy, A.J., Grosse-Kunstleve, R.W., Adams, P.D., Winn, M.D., Storoni, L.C., and Read, R.J. (2007). Phaser crystallographic software. J Appl Crystallogr 40, 658-674.

Murshudov, G.N., Skubak, P., Lebedev, A.A., Pannu, N.S., Steiner, R.A., Nicholls, R.A., Winn, M.D., Long, F., and Vagin, A.A. (2011). REFMAC5 for the refinement of macromolecular crystal structures. Acta Crystallogr D Biol Crystallogr 67, 355-367.

Painter, J., and Merritt, E.A. (2006). Optimal description of a protein structure in terms of multiple groups undergoing TLS motion. Acta Crystallogr D Biol Crystallogr 62, 439-450.

Robert, X., and Gouet, P. (2014). Deciphering key features in protein structures with the new ENDscript server. Nucleic Acids Res 42, W320-324.

Schrödinger LLC. (Version 1.2r3pre). The PyMOL Molecular Graphics System.

Winn, M.D., Ballard, C.C., Cowtan, K.D., Dodson, E.J., Emsley, P., Evans, P.R., Keegan, R.M., Krissinel, E.B., Leslie, A.G., McCoy, A., et al. (2011). Overview of the CCP4 suite and current developments. Acta Crystallogr D Biol Crystallogr 67, 235-242.

Yang, H., Guranovic, V., Dutta, S., Feng, Z., Berman, H.M., and Westbrook, J.D. (2004). Automated and accurate deposition of structures solved by X-ray diffraction to the Protein Data Bank. Acta Crystallogr D Biol Crystallogr 60, 1833-1839.
